# Radiative Warming Glass for High‐Latitude Cold Regions

**DOI:** 10.1002/advs.202414192

**Published:** 2025-01-10

**Authors:** Zhengui Zhou, Rong Liu, Zhen Huang, Bin Hu, Yi Long

**Affiliations:** ^1^ Wuhan National Laboratory for Optoelectronics School of Optical and Electronic Information Huazhong University of Science and Technology Wuhan Hubei 430074 P. R. China; ^2^ Department of Electronic Engineering The Chinese University of Hong Kong Shatin, New Territories Hong Kong SAR 999077 China; ^3^ Shenzhen Huazhong University of Science and Technology Research Institute Shenzhen 518057 China

**Keywords:** energy savings, high‐latitude regions, low‐e glass, radiative warming

## Abstract

Traditional window glazing, with inherently adverse energy‐efficient optical properties, leads to colossal energy losses. Energy‐saving glass requires a customized optical design for different climate zones. Compared with the widely researched radiative cooling technology which is preferable to be used in low‐altitude hot regions; conversely in high‐latitude cold regions, high solar transmittance (*T*_sol_) and low mid‐infrared thermal emissivity (*ε*_MIR_) are the key characteristics of high‐performance radiative warming window glass, while the current low‐emissivity (low‐e) glass is far from ideal. To address this issue, Drude's theory is used to numerically design a near‐ideal film with specified electron density (*n*_e_) and electron mobility (*µ*_e_). The fabricated hydrogen‐doped indium oxide (IHO) could achieve high *T*_sol_ (0.836) and low *ε*_MIR_ (0.117). Energy‐saving simulations further reveal a substantial decrease in annual heating energy consumption up to 6.6% across high‐latitude regions (climate zones 6 to 8), translating to a corresponding reduction in CO_2_ emissions (20.0 kg m^−2^), outperforming 1165 high performance commercial low‐e glass. This radiative warming glass holds the promise of making a significant contribution to sustainable building energy savings specifically for high‐latitude cold regions, advancing the goal of carbon neutrality.

## Graphical abstract

In contrast to widely reported radiative cooling technologies, a radiative warming glass is proposed for high‐latitude cold regions, proving significant energy‐saving potential compared with commercial high‐performance low‐e glass.

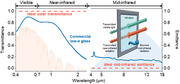

## 1 Introduction

Heating, ventilation, and air conditioning (HVAC) systems, crucial for maintaining suitable indoor environments, contribute to approximately 40% of building energy consumption, resulting in a substantial release of greenhouse gases;^[^ ^1^, ^2^ ^]^ while the primary source of inefficiency is due to building windows, responsible for nearly 60% of heat dissipation.^[^ ^3^, ^4^ ^]^ Various strategies have been proposed to address this issue, notably involving the application of materials with low thermal conductivity such as transparent insulation woods^[^ ^5^ ^]^ and aerogels.^[^ ^6^, ^7^ ^]^ However, these approaches often necessitate installation with centimeters of thickness within enclosed spaces to impart improved heat insulation.^[^ ^8^ ^]^

Recent attention has focused on thin‐film thermal management materials, owing to their engineered optical properties and retrofitability to existing windows.^[^ ^9^, ^10^, ^11^ ^]^ In contrast to widely reported radiative cooling technologies primarily deployed in cooling dominated low latitude regions (Table S1, Supporting Information),^[^ ^12^, ^13^, ^14^, ^15^ ^]^ radiative warming materials for high latitude regions are required to have high solar transmittance (*T*_sol_, 0.3 to 2.5 µm) and low mid‐infrared emissivity (*ε*_MIR_, 4 to 18 µm), which are severely lacking (Table S2, Supporting Information). Normal glass usually exhibits high *T*_sol_ (>0.85), but the high *ε*_MIR_ (≈0.84) results in a significant amount of energy dissipation.^[^ ^16^, ^17^ ^]^ The use of a low‐emissivity (low‐e) coating, particularly an ultra‐thin metal layer like a silver film, is recognized as a significant method to impede radiative heat loss from windows.^[^ ^2^, ^18^ ^]^ The mechanism relies on the high electron concentration for reflecting mid‐infrared radiation, thus achieving a low *ε*_MIR_ (<0.2).^[^ ^19^ ^]^ However, high‐performance commercial silver‐based low‐e glass is often accompanied with compromised *T*_sol_.^[^ ^16^, ^18^ ^]^ Although certain studies explore the application of metal nanowires to improve *T*_sol_,^[^ ^20^ ^]^ challenges persist due to the inherent susceptibility of metal materials to oxidation and their poor adhesion to glass substrates as well as haze.^[^ ^21^, ^22^ ^]^ Additionally, transparent conductive oxides, such as indium tin oxide (ITO), can function as low‐e materials with high visible transmittance (*T*_vis_ > 0.8), but their effectiveness is limited by low near‐infrared transmittance (*T*_NIR_) attributable to a relatively high plasma frequency.^[^ ^23^, ^24^, ^25^ ^]^ Thus, the design and development of high‐performance radiative warming materials with high *T*_sol_ (>0.8) and low *ε*_MIR_ (<0.15) are crucial for further increasing solar energy utilization and reducing radiative heat loss from windows, consequently reducing energy consumption for heating‐dominated high latitude regions.

In this study, we introduce a radiative warming glass employing hydrogen‐doped indium oxide (IHO) to attain the specified electron density (*n*_e_) and electron mobility (*µ*_e_) in accordance with Drude's theory to achieve high *T*_sol_ and low *ε*_MIR_. An optimal IHO glass has been identified, featuring superior *n*_e_ (2.17 × 10^20^ cm^−3^ vs 6.93 × 10^20^ cm^−3^) and *µ*_e_ (98.3 vs 42 cm^2^ V^−1^ s^−1^) compared with commercial ITO glass, which could be further improved with a high *T*_sol_ of 0.836 and a low *ε*_MIR_ of 0.117 with integration of an anti‐reflective overcoat. Energy‐saving simulations further demonstrate a substantial reduction in annual heating energy consumption within specific high latitude regions (climate zones 6 to 8), ranging from 3.4% to 6.6% compared with commercial low‐e glass, corresponding to a CO_2_ emission reduction of 8.9 to 20.0 kg m^−2^. The introduction of this radiative warming glass initiates a new concept for customized energy savings in high latitude regions, offering significant potential in addressing energy shortage challenges and contributing to carbon neutrality.

## 2 Results and Discussions

### 2.1 Ideal Radiative Warming Windows

**Figure 1:**
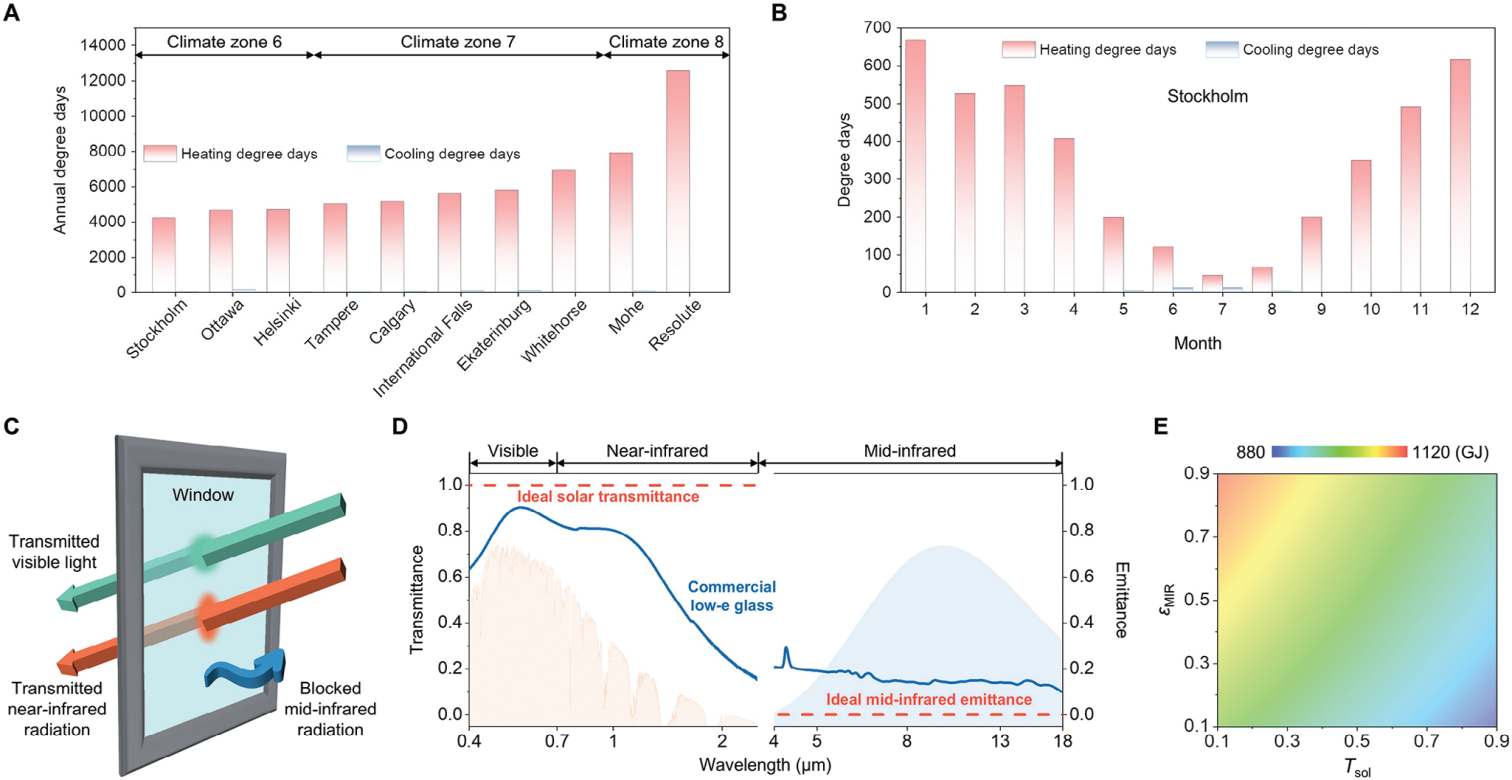
Design principle of the radiative warming window. A) Annual heating and cooling degree days of 10 cities in high latitude regions. B) Heating and cooling degree days over 12 months in Stockholm (Sweden). C) Schematic of the ideal radiative warming window. The window is designed to allow visible light and near‐infrared radiation transmitted, concurrently ensuring effective blocking of mid‐infrared radiation. D) Transmittance and emittance spectra of an ideal radiative warming glass (dashed line) and a commercial ITO low‐e glass (solid line). E) Simulated annual heating energy consumption for a reference mid‐rise apartment building located in Mohe, China. This assessment considers the utilization of windows with varying *T*_sol_ and *ε*_MIR_.

Energy consumption in buildings, specifically for indoor heating and cooling, is commonly quantified through heating and cooling degree days.^[^ ^26^ ^]^ **Figure** **1**A illustrates the annual heating and cooling degree days for 10 cities situated in high latitude regions (Figure S1, Supporting Information), spanning climate zones 6 to 8, including Stockholm (Sweden, climate zone 6), Ottawa (Canada, climate zone 6), Helsinki (Finland, climate zone 6), Tampere (Finland, climate zone 7), Calgary (Canada, climate zone 7), International Falls, MN (USA, climate zone 7), Ekaterinburg (Russia, climate zone 7), Whitehorse (Canada, climate zone 7), Mohe (China, climate zone 8), and Resolute (Canada, climate zone 8). It can be found that heating energy consumption dominates in all these cities. Taking Stockholm as an example, the heating consumption predominates throughout the whole year (Figure 1B), highlighting the necessity for radiative warming materials rather than radiative cooling materials in these high‐latitude zones. An ideal radiative warming window requires higher *T*_vis_ and *T*_NIR_, coupled with lower *ε*_MIR_ to enhance solar transmission and suppress heat emission, while commercial low‐e glass suffers from intrinsically low *T*_NIR_ and compromised *T*_vis_ and *ε*_MIR_ (Figure 1C,D). To validate its potential for energy conservation, we employed the EnergyPlus energy simulation software—an extensively validated whole‐building energy simulator.^[^ ^4^, ^13^ ^]^ The simulation utilized weather data sourced from Mohe (≈53°N), a region characterized by a subarctic climate according to the Köppen classification. A reference mid‐rise apartment building (window area of 234.08 m^2^, and window‐to‐wall ratio of 20.24%), provided by the US Department of Energy (Figure S2, Supporting Information), was used in the simulation in this paper.

As shown in Figure 1E, by systematically changing optical properties of the building window to investigate the effects of varying *T*_sol_ and *ε*_MIR_ on annual energy consumption, improving *T*_sol_ and reducing *ε*_MIR_ of building windows lead to a notable decrease in heating energy consumption. For example, employing normal glass with *T*_sol_ of 0.78 and *ε*_MIR_ of 0.84 results in a heating energy consumption of 988.4 GJ for the entire apartment building (Figure S3, Supporting Information). Substituting the normal glass with commercial low‐e glass (*T*_sol_ of 0.63 and *ε*_MIR_ of 0.1) decreases heating energy consumption to 944.1 GJ, presenting an approximate 4.5% energy‐saving potential. We further calculated the heating energy consumption when employing window glass with a nearly ideal *T*_sol_ and unchanged *ε*_MIR_ (*T*_sol_ of 0.99 and *ε*_MIR_ of 0.1). The simulation results indicate that heating energy consumption could be further reduced to 854 GJ, thereby amplifying the energy‐saving potential to 9.5% compared to commercial low‐e glass. These assessments demonstrate that window glass with increased *T*_sol_ and decreased *ε*_MIR_ can significantly reduce heating energy consumption compared to commercial low‐e glass.

### 2.2 Design Principle of Materials

As a traditional low‐e material, commercial ITO glass falls short of perfection due to its relatively low *T*_NIR_ (≈0.7, Figure 1D), due to the high plasma frequency.^[^ ^25^ ^]^ To design and fabricate a material for radiative warming, we delved into understanding the relationship between the material's electrical properties and its spectral reflectance based on the classical Drude's model.^[^ ^27^ ^]^ Assuming that the free electrons *e* within the material undergo accelerated motion under the electric field and collide with stationary atoms, we can calculate the dielectric function of the material as follows:

(1) $$\varepsilon \;\left(\omega \right)=1-\frac{\omega_{p}^{2}}{\omega \left(\omega +i\tau^{-1}\right)}$$

where ω_p_ denotes the plasma frequency, which is determined by the equation $\omega_{p}^{2}=\frac{n_{e}e^{2}}{m_{e}\varepsilon_{0}}$. In this equation, *e* represents the absolute charge of an electron, *m*_e_ is the electron mass, and the electron density is denoted as *n*_e_. The parameter *τ* is related to the electron mobility (*µ*_e_), where $\tau =\frac{m_{e}\mu_{e}}{e}$. Here, *ε*₀ represents the vacuum permittivity, equal to 8.854 × 10⁻¹^2^ F m⁻¹. Thus, ω_p_ is influenced by *n*_e_, and is independent of *µ*_e_.

The dielectric function can also be expressed in terms of the refractive index (*n*) and extinction coefficient (*κ*):

(2) $$\varepsilon \;\left(\omega \right)={\left(n\left(\omega \right)+i\kappa \left(\omega \right)\right)}^{2}$$

**Figure 2:**
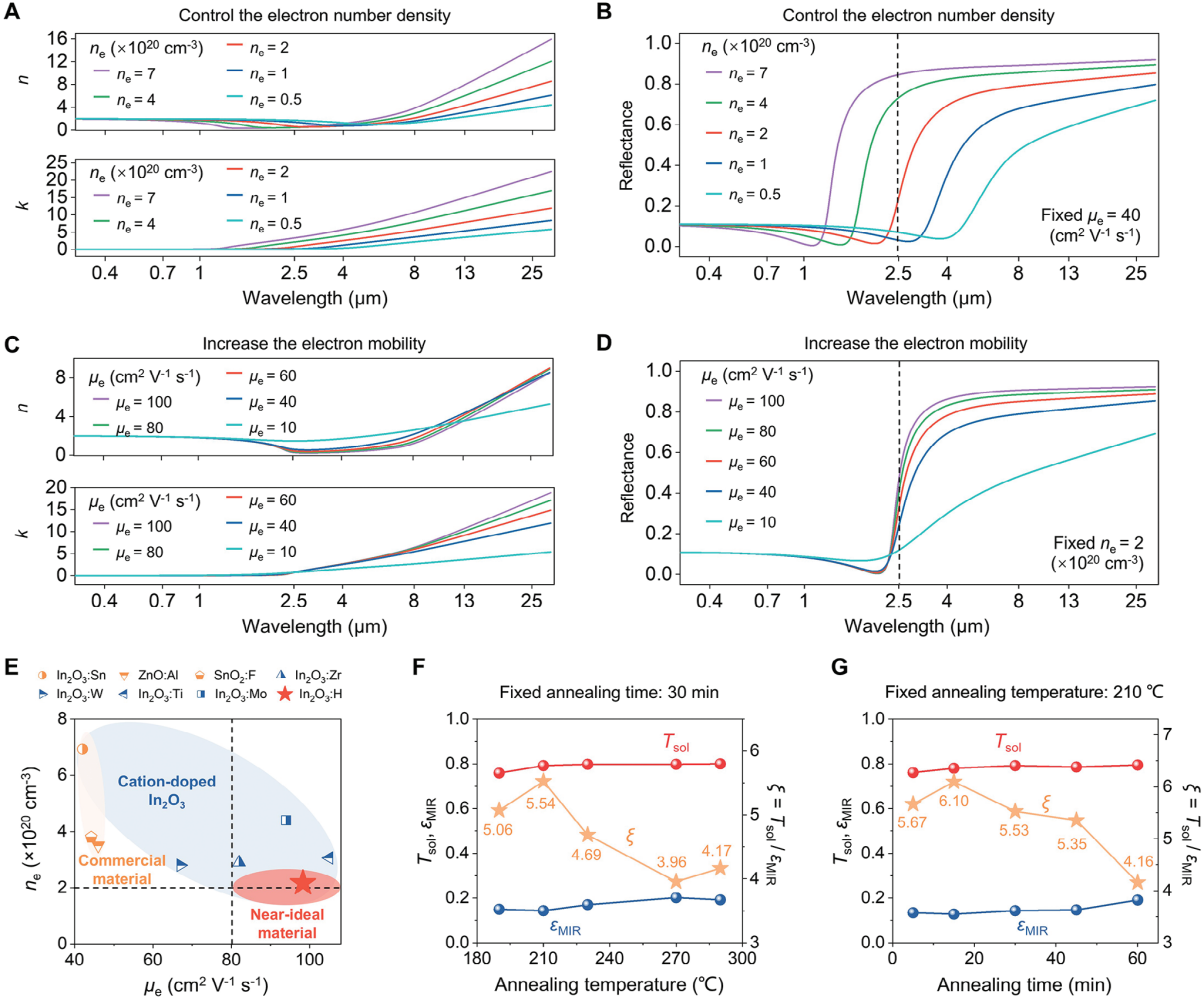
Design principle of materials with optimal spectral characteristics. A) Calculated refractive index *n* and extinction coefficient *κ* at different *n*_e_ with fixing *µ*_e_. B) Calculated reflectance spectra for varying *n*_e_ with fixing *µ*_e_. C) Calculated refractive index *n* and extinction coefficient *κ* at different *µ*_e_ with fixing *n*_e_. D) Calculated reflectance spectra for varying *µ*_e_ with fixing *n*_e_. E) Comparison of *n*_e_ and *µ*_e_ across different typical oxides including commercial conductive materials and cation‐doped In_2_O_3_,^[^ ^19^, ^28^, ^29^, ^30^, ^31^, ^35^ ^]^ with near‐ideal materials denoted by red regions. F) Measured *T*_sol_, *ε*_MIR_ and corresponding *ξ* = *T*_sol_/*ε*_MIR_ at different post‐annealing temperatures with fixing annealing time. G) Measured *T*_sol_, *ε*_MIR_ and corresponding *ξ* values at different post‐annealing time with fixing annealing temperature.

Hence, the relationship between the refractive index *n* and the extinction coefficient *κ* for the incident wave frequency (*ω*) can be established by combining Equations 1 and 2,^[^ ^19^ ^]^ and the spectral reflectance (*ρ*) can be investigated based on the relationship $\rho =\frac{{\left(n-1\right)}^{2}+\kappa^{2}}{{\left(n+1\right)}^{2}+\kappa^{2}}$. By fixing *µ*_e_ of 40 cm^2^ V^−1^ s^−1^, an increase in *n*_e_ from 0.5 × 10^20^ to 7 × 10^20^ cm^−3^ leads to an increase of *κ* in both NIR and MIR range, while *n* decreases in NIR and increases in MIR range (**Figure** **2**A), which leading to an increase of both NIR and MIR reflectance (*R*_NIR_ and *R*_MIR_) (Figure 2B), and thus decreasing *T*_NIR_ and *ε*_MIR_. Moreover, investigations extending *n*_e_ beyond the range of 0.5 × 10^20^ to 7 × 10^20^ cm^−3^ further confirm these observed trends (Figure S4, Supporting Information). Notably, since *n*_e_ significantly affects reflectance across the entire solar spectrum, *n*_e_ of 2 × 10^20^ cm^−3^ yields the lowest *R*_sol_ (Figure S5, Supporting Information). We further explored the effect of *µ*_e_ on spectral reflectance based on the corresponding *n* and *κ* by fixing *n*_e_ of 2 × 10^20^ cm^−3^ (Figure 2C). Increasing the material's *µ*_e_ results in a significant increase in *R*_MIR_, while *R*_NIR_ has negligible changes, suggesting higher *µ*_e_ (>80 cm^2^ V^−1^ s^−1^) is preferred (Figure 2D).

Considering the influence of both *n*_e_ and *µ*_e_ on spectral reflectance, it is reasonable to customize the material's NIR and MIR spectra by fine‐tuning *n*_e_ and *µ*_e_. In the case of commercial materials such as Sn‐doped In_2_O_3_ (ITO), which possesses a high *n*_e_ of 6.93 × 10^20^ cm^−3^ and low *µ*_e_ of 42 cm^2^ V^−1^ s^−1^, alternative elements rather than tin can be doped into In_2_O_3_ to achieve the desired *n*_e_ (≈2 × 10^20^ cm^−3^) and higher *µ*_e_ (>80 cm^2^ V^−1^ s^−1^). Metals such as titanium (Ti),^[^ ^28^ ^]^ zirconium (Zr),^[^ ^29^ ^]^ molybdenum (Mo),^[^ ^30^ ^]^ and tungsten (W)^[^ ^31^ ^]^ have been reported to attain high *µ*_e_, but their *n*_e_ does not meet the specified requirement (Figure 2E). Additionally, their preparation often requires a high‐temperature environment (≥300 °C) and involves relatively stringent processing methods.^[^ ^32^ ^]^ Herein, we employed a mild magnetron sputtering doping with hydrogen as an alternative approach (Figure S6, Supporting Information),^[^ ^32^, ^33^, ^34^ ^]^ and demonstrated it as an energy‐efficient radiative warming window.

The post‐annealing treatment has been confirmed as an effective step in enhancing the crystallinity of the as‐deposited IHO film after a radio frequency (RF) magnetron sputtering process.^[^ ^33^ ^]^ To determine the optimal annealing conditions, we measured the spectra of various annealed samples using a UV–VIS–NIR spectrophotometer and a Fourier transform infrared (FTIR) spectrometer, respectively (Figure S7, Supporting Information). *T*_sol_ and *ε*_MIR_ were calculated based on the measured spectra, and spectral selectivity (*ξ* = *T*_sol_/*ε*_MIR_) was introduced to evaluate the radiative warming ability of these annealed samples.^[^ ^19^, ^36^ ^]^ By fixing post‐annealing time of 30 min (Figures 2F and S7A,B, Supporting Information), as the post‐annealing temperature gradually increased from 190 °C to 210 °C, *T*_sol_ increased from 0.759 to 0.792, while *ε*_MIR_ basically unchanged (0.150 vs 0.143), leading to improved *ξ* (5.06 vs 5.54). Further increasing post‐annealing temperature to 290 °C decreased *ξ* (4.17) due to improved *ε*_MIR_ (0.192) and relatively constant *T*_sol_ (0.800). Thus, an optimal annealing temperature of 210 °C was selected as the optimal condition. Additionally, the effect of post‐annealing time was also investigated by fixing annealing temperature of 210 °C (Figures 2G and S7C,D, Supporting Information). Annealing treatment of 15 min was optimal to achieve a high *ξ* of 6.10, and longer increasing time up to 60 min resulted in decreased *ξ* (4.16).

The prepared IHO glass under optimal annealing condition (210 °C, 15 min) has a high *T*_sol_ of up to 0.780 and low *ε*_MIR_ of 0.128, featuring *n*_e_ of 2.17 × 10^20^ cm^−3^ and *µ*_e_ of 98.3 cm^2^ V^−1^ s^−1^, close to the required values mentioned earlier (*n*_e_ ≈ 2 × 10^20^ cm^−3^, *µ*_e_ > 80 cm^2^ V^−1^ s^−1^) shown in Figure 2E. This IHO glass demonstrates electrical conductivity (≈10 Ω/□) similar to that of commercial ITO glass (Figure S8, Supporting Information), enabling rapid temperature increase with a small voltage (Figure S9, Supporting Information). The durability of IHO glass was primarily evaluated, with consistent *T*_sol_ and *ε*_MIR_ observed after three months of outdoor exposure (Figure S10, Supporting Information).

### 2.3 Characterizations of Radiative Warming Glass

**Figure 3:**
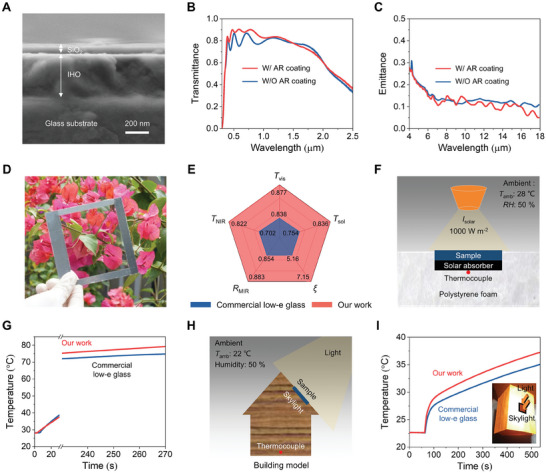
Characterizations of radiative warming glass incorporating IHO glass with anti‐reflective (AR) SiO_2_ coating. A) Cross‐sectional SEM image of radiative warming glass. B) Measured transmittance of IHO glass with and without AR overcoat. C) Measured emittance of IHO glass with and without AR overcoat. D) Real image of fabricated radiative warming glass with metal frame. E) A comprehensive comparison between radiative warming glass and commercial ITO low‐e glass, including *T*_vis_, *T*_NIR_, *T*_sol_, *R*_MIR_ and *ξ*. F) Schematic illustrating the setup for a real‐time temperature test, wherein the radiative warming glass is applied above a solar absorber. The testing environment is maintained at a constant temperature of 28 °C and a relative humidity of 50%. A thermocouple is attached to the backside of the solar absorber. G) Temperature measurements during the real‐time test for the solar absorber with radiative warming glass and commercial ITO low‐e glass, respectively. H) Schematic illustrating the setup for a real‐time temperature test, wherein the radiative warming glass is applied on a model building to serve as a skylight. The testing environment is maintained at a constant temperature of 22 °C and a relative humidity of 50%. A thermocouple is attached to the bottom of the model building. I) Temperature measurements during the real‐time test for the model building with radiative warming glass and commercial ITO low‐e glass, respectively. The inset shows a real photo of the setup.

Based on the optimal IHO glass, we applied an anti‐reflective (AR) layer to its surface to form a radiative warming glass. General AR coatings often employ complex multilayer structures or nanostructure arrays to minimize interface reflections.^[^ ^37^, ^38^, ^39^, ^40^, ^41^ ^]^ Herein, silicon dioxide (SiO_2_) was deposited on the surface of the IHO glass, which exhibits a hydrophilic surface (Figure S11, Supporting Information). The cross‐sectional morphology of the radiative warming glass was examined using a scanning electron microscope (SEM) in **Figure** **3**A, showing a SiO_2_ coating thickness of approximately 65 nm on the IHO underlayer (≈370 nm). This ultrathin SiO_2_ coating could reduce interface reflections (Figure S12, Supporting Information), thereby improving *T*_sol_ (0.836 vs 0.780) of the IHO glass (Figure 3B). Importantly, the ultrathin SiO_2_ layer had a negligible effect on the low *ε*_MIR_ of IHO glass (0.117 vs 0.128) (Figure 3C).

The actual photo reveals that the radiative warming glass possesses high visible transparency, allowing a vivid, unobstructed view of the flowers located behind it (Figure 3D). To demonstrate its radiative warming capabilities, Figure 3E provides a comprehensive comparison of the optical properties between the radiative warming glass and commercial ITO low‐e glass. The radiative warming glass exhibits higher *T*_sol_ (0.836 vs 0.754), higher *T*_vis_ (0.877 vs 0.838), higher *T*_NIR_ (0.822 vs 0.702), higher *R*_MIR_ (0.883 vs 0.854), and higher *ξ* (7.15 vs 5.16) compared to commercial ITO low‐e glass. Detailed parameters are listed in Table S3 (Supporting Information).

We further monitored the real‐time temperature of a solar absorber (silicon wafer) with a glass sample (radiative warming glass or commercial ITO low‐e glass) positioned above it to demonstrate the capability for solar energy transmission.^[^ ^16^, ^42^ ^]^ As shown in Figure 3F, both the glass sample and the underlying solar absorber were enclosed by a polystyrene foam board to minimize conductive and convective heat dissipation during the test. The testing environment maintained a constant ambient temperature (*T*_amb_) of 28 °C, with a relative humidity (RH) of approximately 50%. Upon illuminating the surfaces of both glass samples with a solar simulator (1000 W m^−2^), the temperature measured by the thermocouple underneath the solar absorber sharply increases from an initial state of ≈28 °C (Figure 3G). After 240 s, the temperature rise decelerates. Notably, the solar absorber temperature reaches ≈75 °C when using commercial low‐e glass. In contrast, with radiative warming glass, the solar absorber temperature is ≈80 °C. This temperature difference of ≈5 °C suggests that radiative warming glass has an improved passive heating capability compared to commercial low‐e glass. The steady‐state temperature simulations suggest consistency with the experimental results, as the higher solar irradiance and reduced non‐radiative heat transfer would contribute to a larger temperature difference (Figure S13, Supporting Information).

Furthermore, a model building with a skylight was positioned beneath a solar simulator to evaluate the indoor warming performance of radiative warming glass compared to commercial low‐e glass (Figure 3H). Thermocouples were placed at the base of the model building to monitor the indoor temperature. The ambient temperature was maintained at 22 °C, with a relative humidity of 50%. As shown in Figure 3I, the solar simulator directly illuminated the skylight. The indoor temperature of the model building increased more rapidly when radiative warming glass was employed compared to commercial low‐e glass. After 500 s, the indoor temperature of the model building with radiative warming glass was 2.1 °C higher than that with commercial low‐e glass. This indoor temperature increase is attributed to increased solar energy income and reduced heat loss compared with commercial low‐e glass. Additionally, the simulation conducted on an apartment building demonstrated that the radiative warming glass achieved a higher temperature than the commercial low‐e glass, proving its potential for effective radiative warming in actual building applications (Figure S14, Supporting Information).

### 2.4 Energy Saving Simulation

**Figure 4:**
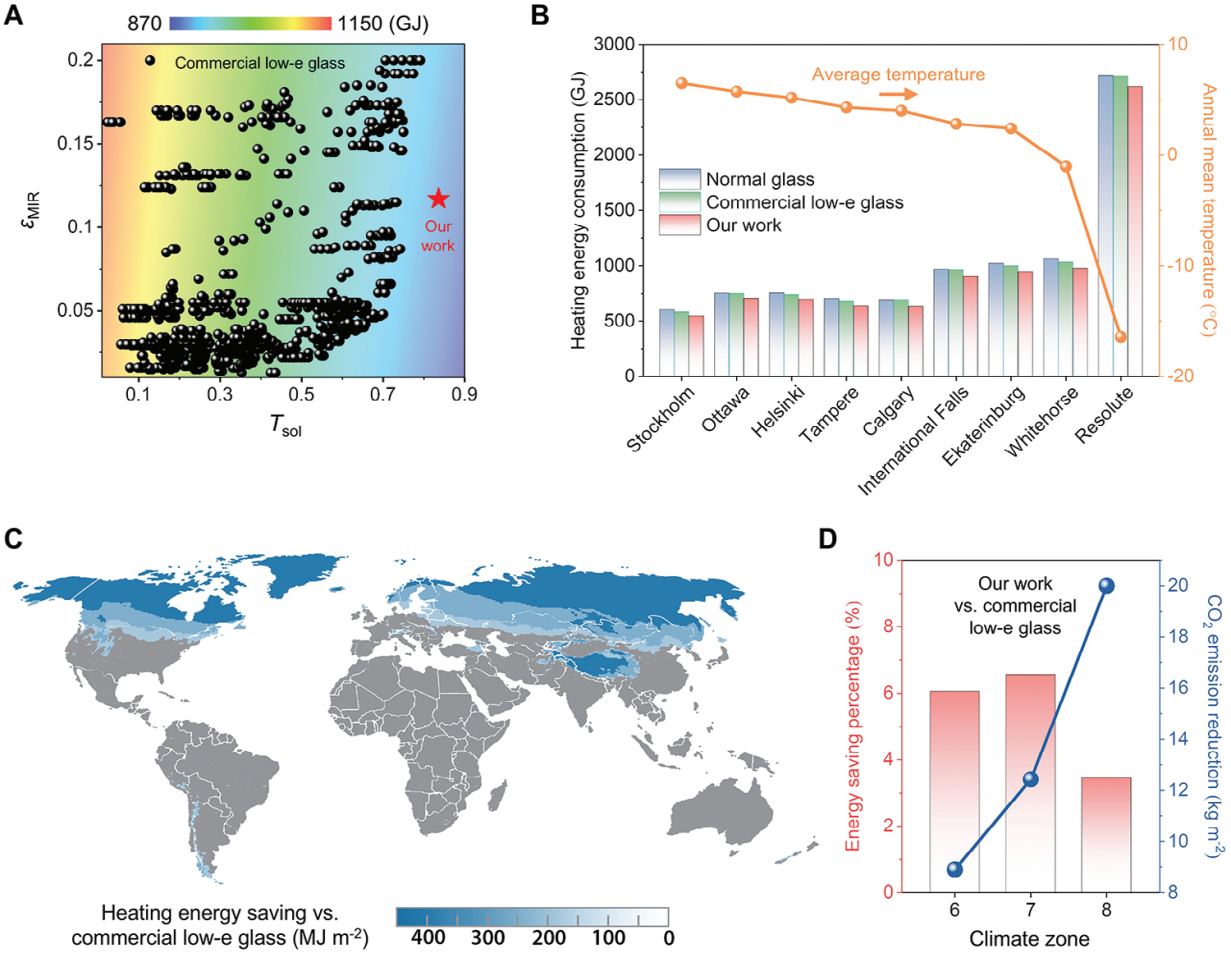
Simulation of heating energy savings and CO_2_ emission reduction for radiative warming glass. A) Comparison of heating energy savings between commercial silver‐based low‐e glass and our radiative warming glass. The black point represents 1165 types of commercial low‐e glass for reference. The detailed parameters can be found in Table S4 (Supporting Information). B) Comparison of heating energy consumption for a mid‐rise apartment building in 9 representative cities when utilizing normal glass, commercial low‐e glass, and our radiative warming glass as windows. C) Annually estimated heating energy‐saving potential across high latitude regions by the application of radiative warming glass, as compared to commercial low‐e glass. D) Annually estimated energy‐saving percentage and corresponding reduction in CO_2_ emissions in climate zones 6 to 8, utilizing radiative warming glass in comparison to commercial low‐e glass.

To demonstrate the energy‐saving potential of the fabricated radiative warming glass (Figure 1E), 1165 types of high‐performance commercial silver‐based low‐e glass (by setting the limit of *ε*_MIR_ of 0.2) were sourced from the International Glazing Database (Table S4, Supporting Information) and their *T*_sol_ and *ε*_MIR_ were compared with the IHO‐based radiative warming glass (**Figure** **4**A), suggesting that the fabricated radiative warming glass outperform all 1165‐commercial low‐e glass with higher *T*_sol_ and comparable *ε*_MIR_.

We further conducted an energy‐saving simulation to evaluate the heating energy‐saving potential of the radiative warming glass located in high‐latitude regions which represented climate zones ranging from 6 to 8, including Stockholm (Sweden, climate zone 6), Ottawa (Canada, climate zone 6), Helsinki (Finland, climate zone 6), Tampere (Finland, climate zone 7), Calgary (Canada, climate zone 7), International Falls, MN (USA, climate zone 7), Ekaterinburg (Russia, climate zone 7), Whitehorse (Canada, climate zone 7), and Resolute (Canada, climate zone 8). The heating energy consumption for the entire building was assessed using normal glass, commercial low‐e glass, and radiative warming glass (Table S5, Supporting Information). Figure 4B shows that as the annual average ambient temperature gradually decreases from 6.5 to −16.4 °C from climate zones 6 to 8, the heating energy consumption in these cities significantly increases. Among these cities, the heating energy consumption of the apartment building is lower when using commercial low‐e glass compared to normal glass. For example, Whitehorse, a city located in the Yukon Territory of Canada, characterized by long and harsh winters with an annual average ambient temperature of −1 °C, gives a marginal decrease in heating energy consumption from 1061.37 GJ to 1033.08 GJ, while radiative warming glass could further reduce to 974.55 GJ. The radiative warming glass demonstrate to be more energy‐efficient compared to commercial low‐e glass in high latitude regions.

Figure 4C shows the energy‐saving potential map of radiative warming glass compared to commercial low‐e glass across climate zones 6 from 8. Radiative warming glass can significantly reduce average annual heating energy consumption, ranging from 177.9 to 399.6 MJ m^−2^, representing 3.4% to 6.6% of the total energy consumption (Figure 4D), including climate zone 6 (6.1%), climate zone 7 (6.6%), and climate zone 8 (3.4%). Moreover, we converted the energy savings of this mid‐rise building into annual natural gas savings. The use of radiative warming glass can save 4.6 m^3^ m^−2^ (climate zone 6), 6.4 m^3^ m^−2^ (climate zone 7), and 10.3 m^3^ m^−2^ (climate zone 8) of natural gas annually (Figure S14, Supporting Information), corresponding to annual CO_2_ emission reduction of 8.9 kg m^−2^ (climate zone 6), 12.4 kg m^−2^ (climate zone 7), 20.0 kg m^−2^ (climate zone 8), respectively (Figure 4D). Additionally, a preliminary cost analysis solely based on the materials cost indicates that this radiative warming glass could provide a good option compared with commercial low‐e glass (Table S6, Supporting Information).

## 3 Conclusion

In summary, by tuning electron density and mobility, a high‐performance radiative warming glass for sustainable building in high latitude regions has been numerically designed and experimentally fabricated. An optimized IHO layer with desired electron density (2.17 × 10^20^ cm^−3^) and mobility (98.3 cm^2^ V^−1^ s^−1^) exhibits superior optical properties, giving high *T*_sol_ (0.836) and low *ε*_MIR_ (0.117). Simulation results demonstrate a significant energy‐saving potential in annual heating energy consumption, giving up to 3.4% to 6.6% energy savings compared with commercial low‐e glass, corresponding to a CO_2_ emission reduction ranging from 8.9 to 20.0 kg m^−2^. Compared with much investigated radiative cooling materials, this newly fabricated radiative warming glass could emerge as a sustainable solution for high latitude regions, contributing positively to global carbon neutrality and sustainability.

## 4 Experimental Section

### Fabrication of the Radiative Warming Glass

The production of the IHO involved employing a radio frequency (RF) magnetron sputtering process. The sputtering process utilized a high‐purity ceramic In_2_O_3_ target with a high‐purity level of 99.99% (Zhongnuo Advanced Material Technology Co., Ltd). The soda‐lime glass was cleaned by ethanol and dried before magnetron sputtering. The vacuum pressure within the RF magnetron sputtering system (JCP‐600M4, Beijing Technol Science Co., Ltd) was first reduced to 10^−4^ Pa. Then, a gas mixture comprising 95% argon and 5% hydrogen were introduced into the sputtering vacuum chamber. After sputtering at a power of 100 W for 30 min at room temperature, the as‐deposited IHO film was annealed at different temperatures in a nitrogen atmosphere.

An anti‐reflective SiO_2_ coating was also fabricated using the RF sputtering method, with the utilization of a SiO_2_ target (Zhongnuo Advanced Material Technology Co., Ltd) within the RF magnetron sputtering system (JCP‐600M4, Beijing Technol Science Co., Ltd). The vacuum pressure was first reduced to 1.2 × 10^−3^ Pa. Subsequently, argon gas was introduced into the chamber to attain a pressure of 0.6 Pa, followed by the introduction of oxygen to achieve a pressure of 0.7 Pa. Finally, the SiO_2_ coating was deposited onto the surface of IHO glass using RF sputtering with a power of 200 W for a controlled duration of 15 min.

### Characterizations

Cross‐sectional morphology was investigated using a scanning electron microscope (FEI Nova Nano SEM 450). Electrical properties were assessed through Hall effect measurement (HMS‐7000, Ecopia), employing a magnetic field of 0.55 T and an operating current of 15 mA. Sheet resistance was measured using a four‐probe resistivity tester (RTS‐3). Solar transmittance and reflectance within the 0.3 to 2.5 µm range were characterized by a UV‐VIS‐NIR spectrophotometer (Shimadzu UV‐3600 Plus) with an integrating sphere attachment (ISR‐603). Mid‐infrared emittance was determined using a Fourier‐transform infrared spectrometer (Nicolet is50) equipped with a gold integrated sphere attachment. Contact angles were measured using a contact angle meter (SL200B, Kino). Real‐time temperatures were monitored using thermocouples (OMEGA TT‐K‐36‐SLE) connected to a data logger (PICO TC‐08). The building model in the experiment had dimensions of approximately 25 cm × 18 cm × 15 cm, and the skylight measured about 5 cm × 5 cm. Additionally, a humidity thermometer (CENTER SE‐310) measured the temperature and humidity of the environment. The commercial ITO low‐e glass was obtained from Foshan Shi Yuan Jing Mei Glass Co., Ltd.

### Calculating from the Spectrum

The following equations were utilized to calculate the integral solar transmittance (*T*_sol_), solar reflectance (*R*_sol_), visible transmittance (*T*_vis_), visible reflectance (*R*_vis_), near‐infrared transmittance (*T*_NIR_) and mid‐infrared emissivity (*ε*_MIR_), as expressed by Equations (3), (4), (5), (6), (7), (8), respectively.^[^ ^4^ ^]^

(3) $$T_{\mathrm{sol}}=\frac{\int_{0.3}^{2.5}d\lambda \cdot t\left(\lambda \right)\cdot I_{\mathrm{AM}.1.5}\left(\lambda \right)}{\int_{0.3}^{2.5}d\lambda \cdot I_{\mathrm{AM}.1.5}\left(\lambda \right)}\;$$

(4) $$R_{\mathrm{sol}}=\frac{\int_{0.3}^{2.5}d\lambda \cdot r\left(\lambda \right)\cdot I_{\mathrm{AM}.1.5}\left(\lambda \right)}{\int_{0.3}^{2.5}d\lambda \cdot I_{\mathrm{AM}.1.5}\left(\lambda \right)}\;$$

(5) $$T_{\mathrm{vis}}=\frac{\int_{0.4}^{0.7}d\lambda \cdot t\left(\lambda \right)\cdot I_{\mathrm{AM}.1.5}\left(\lambda \right)}{\int_{0.4}^{0.7}d\lambda \cdot I_{\mathrm{AM}.1.5}\left(\lambda \right)}\;$$

(6) $$R_{\mathrm{vis}}=\frac{\int_{0.4}^{0.7}d\lambda \cdot r\left(\lambda \right)\cdot I_{\mathrm{AM}.1.5}\left(\lambda \right)}{\int_{0.4}^{0.7}d\lambda \cdot I_{\mathrm{AM}.1.5}\left(\lambda \right)}\;$$

(7) $$T_{\mathrm{NIR}}=\frac{\int_{0.7}^{2.5}d\lambda \cdot t\left(\lambda \right)\cdot I_{\mathrm{AM}.1.5}\left(\lambda \right)}{\int_{0.7}^{2.5}d\lambda \cdot I_{\mathrm{AM}.1.5}\left(\lambda \right)}\;$$

(8) $$\varepsilon_{\mathrm{MIR}}=\frac{\int_{4}^{18}d\lambda \cdot \left(1-r\left(\lambda \right)\right)\cdot I_{\mathrm{BB}}\left(T,\lambda \right)}{\int_{4}^{18}d\lambda \cdot I_{\mathrm{BB}}\left(T,\lambda \right)}\;$$

where *I*_AM1.5_(*λ*) represents the solar illumination of the standard AM 1.5 spectrum and *I*_BB_ (*T*, *λ*) denotes the spectral radiance of a blackbody at temperature *T*. The variables *t* (*λ*) and *r* (*λ*) correspond to the measured transmittance and reflectance within the relevant wavelength range, respectively. According to Kirchhoff's law, the sum of mid‐infrared reflectance, transmittance, and absorption equals 1. Notably, *T*_MIR_ of the glass sample is assessed to be 0.

### Energy Saving Simulation

To assess the impact of *T*_sol_ and *ε*_MIR_ on heating energy consumption, simulations were used using the EnergyPlus software, a widely recognized whole‐building energy simulator.^[^ ^4^, ^13^ ^]^ These simulations aimed to understand how different *T*_sol_ and *ε*_MIR_ characteristics impact space heating loads and demonstrate the significance of the radiative warming window. The building model, obtained from the US Department of Energy, included a window area of 234.08 m^2^ and a window‐to‐wall ratio of 20.24%. With a total area of 2350.96 m^2^ and a net conditional area of 2117.98 m^2^, the model reflected real‐world scenarios. The optical properties for normal glass and commercial low‐e glass used in the simulation were sourced from reference,^[^ ^4^ ^]^ which can be found in Table S5 (Supporting Information). Mohe, China, is chosen as a typical high‐latitude region for the simulation. Additionally, various regions were simulated worldwide to predict heating energy savings, including Stockholm (Sweden, climate zone 6), Ottawa (Canada, climate zone 6), Helsinki (Finland, climate zone 6), Tampere (Finland, climate zone 7), Calgary (Canada, climate zone 7), International Falls, MN (USA, climate zone 7), Ekaterinburg (Russia, climate zone 7), Whitehorse (Canada, climate zone 7), and Resolute (Canada, climate zone 8). This analysis focused on building energy savings per unit area, which were calculated based on window glass area. For natural gas, a conversion factor of 25.866 m^3^ GJ^−1^ was applied to determine natural gas volume savings. CO_2_ emission reduction was calculated by converting natural gas savings using the relevant conversion factor:^[^ ^43^ ^]^ 1 m^3^ natural gas = 1.935 kg CO_2_.

## Conflict of Interest

The authors declare no conflict of interest.

## Author Contributions

Conceptualization: Z.Z., Y.L., and B.H.; methodology: Z.Z., R.L., and Z.H.; investigation: Z.Z., Z.H., and B.H.; visualization: Z.Z., R.L., and B.H.; supervision: Y.L. and B.H.; writing‐original draft: Z.Z.; writing‐review & editing: Y.L. and B.H. All authors discussed the results and contributed to the final version of the manuscript.

## Supporting information



Supporting Information

## Data Availability

The data that support the findings of this study are available from the corresponding author upon reasonable request.

## References

[1] Y. Ke , C. Zhou , Y. Zhou , S. Wang , S. H. Chan , Y. Long , Adv. Funct. Mater. 2018, 28, 1800113.

[2] B. P. Jelle , S. E. Kalnæs , T. Gao , Energy Build. 2015, 96, 329.

[3] S. Grynning , A. Gustavsen , B. Time , B. P. Jelle , Energy Build. 2013, 61, 185.

[4] S. Wang , T. Jiang , Y. Meng , R. Yang , G. Tan , Y. Long , Science 2021, 374, 1501. 34914526 10.1126/science.abg0291

[5] R. Mi , T. Li , D. Dalgo , C. Chen , Y. Kuang , S. He , X. Zhao , W. Xie , W. Gan , J. Zhu , J. Srebric , R. Yang , L. Hu , Adv. Funct. Mater. 2019, 30, 1907511.

[6] E. Abraham , V. Cherpak , B. Senyuk , J. B. ten Hove , T. Lee , Q. Liu , I. I. Smalyukh , Nat. Energy 2023, 8, 381.

[7] U. Berardi , Appl. Energy 2015, 154, 603.

[8] B. P. Jelle , Energy Build. 2011, 43, 2549.

[9] S. Fan , Joule 2017, 1, 264.

[10] X. Li , W. Xie , C. Sui , P.‐C. Hsu , ACS Mater. Lett. 2020, 2, 1624.

[11] Z. Zhou , Y. Fang , X. Wang , E. Yang , R. Liu , X. Zhou , Z. Huang , H. Yin , J. Zhou , B. Hu , Nano Energy 2022, 93, 106865.

[12] T. Li , Y. Zhai , S. He , W. Gan , Z. Wei , M. Heidarinejad , D. Dalgo , R. Mi , X. Zhao , J. Song , J. Dai , C. Chen , A. Aili , A. Vellore , A. Martini , R. Yang , J. Srebric , X. Yin , L. Hu , Science 2019, 364, 760. 31123132 10.1126/science.aau9101

[13] K. Lin , S. Chen , Y. Zeng , T. C. Ho , Y. Zhu , X. Wang , F. Liu , B. Huang , C. Y. Chao , Z. Wang , C. Y. Tso , Science 2023, 382, 691. 37943925 10.1126/science.adi4725

[14] X. Zhao , T. Li , H. Xie , H. Liu , L. Wang , Y. Qu , S. C. Li , S. Liu , A. H. Brozena , Z. Yu , J. Srebric , L. Hu , Science 2023, 382, 684. 37943922 10.1126/science.adi2224

[15] Z. Zhou , X. Wang , Y. Ma , B. Hu , J. Zhou , Cell Rep. Phys. Sci. 2020, 1, 100231.

[16] F. Giovannetti , S. Föste , N. Ehrmann , G. Rockendorf , Sol. Energy 2014, 104, 52.

[17] Y. Ke , Y. Li , L. Wu , S. Wang , R. Yang , J. Yin , G. Tan , Y. Long , ACS Energy Lett. 2022, 7, 1758.

[18] D.‐C. Tsai , Z.‐C. Chang , B.‐H. Kuo , E.‐C. Chen , Y.‐L. Huang , T.‐J. Hsieh , F.‐S. Shieu , Ceram. Int. 2020, 46, 7991.

[19] V. D. L. M. Simonis , F. C. J. Hoogendoorn , Sol. Energy Mater. 1979, 1, 221.

[20] H. Hu , S. Wang , X. Feng , M. Pauly , G. Decher , Y. Long , Chem. Soc. Rev. 2020, 49, 509. 31845689 10.1039/c9cs00382g

[21] S. Lin , H. Wang , X. Zhang , D. Wang , D. Zu , J. Song , Z. Liu , Y. Huang , K. Huang , N. Tao , Z. Li , X. Bai , B. Li , M. Lei , Z. Yu , H. Wu , Nano Energy 2019, 62, 111.

[22] H. Hu , S. Wang , Y. Meng , G. Liu , M. Li , T. D. Vu , Y. Long , Adv. Mater. Technol. 2021, 7, 2100824.

[23] D. Alonso‐Álvarez , L. Ferre Llin , A. Mellor , D. J. Paul , N. J. Ekins‐Daukes , Sol. Energy 2017, 155, 82.

[24] K. Sun , X. Tang , C. Yang , D. Jin , Ceram. Int. 2018, 44, 19597.

[25] S. H. Brewer , S. Franzen , J. Phys. Chem. B 2002, 106, 12986.

[26] X. Li , B. Sun , C. Sui , A. Nandi , H. Fang , Y. Peng , G. Tan , P.‐C. Hsu , Nat. Commun. 2020, 11, 6101. 33257693 10.1038/s41467-020-19790-x PMC7705009

[27] Z. M. Zhang , Nano/Microscale Heat Transfer, McGraw‐Hill, New York 2007.

[28] R. Hashimoto , Y. Abe , T. Nakada , Appl. Phys. Express 2008, 1, 015002.

[29] T. Koida , M. Kondo , J. Appl. Phys. 2007, 101, 063705.

[30] N. Yamada , T. Tatejima , H. Ishizaki , T. Nakada , Jpn. J. Appl. Phys. 2006, 45, L1179.

[31] M. Yang , J. Feng , G. Li , Q. Zhang , J. Cryst. Growth 2008, 310, 3474.

[32] W. Tong , E. Yang , Y. Pang , H. Yang , X. Qian , R. Yang , B. Hu , J. Dong , X. Zhang , Laser Photonics Rev. 2023, 17, 2201032.

[33] C. Ge , E. Yang , X. Zhao , C. Yuan , S. Li , C. Dong , Y. Ruan , L. Fu , Y. He , X. Zeng , H. Song , B. Hu , C. Chen , J. Tang , Small 2022, 18, 2203677. 10.1002/smll.202203677 36148851

[34] Y. Magari , T. Kataoka , W. Yeh , M. Furuta , Nat. Commun. 2022, 13, 1078. 35228522 10.1038/s41467-022-28480-9 PMC8885685

[35] O. K. C. Agashe , J. Hüpkes , U. Zastrow , B. Rech , M. Wuttig , J. Appl. Phys. 2004, 95, 1911.

[36] Y. Li , C. Lin , Z. Wu , Z. Chen , C. Chi , F. Cao , D. Mei , H. Yan , C. Y. Tso , C. Y. H. Chao , B. Huang , Adv. Mater. 2020, 33, 2005074. 10.1002/adma.202005074 33241608

[37] D. Berman , S. Guha , B. Lee , J. W. Elam , S. B. Darling , E. V. Shevchenko , ACS Nano 2017, 11, 2521. 28139905 10.1021/acsnano.6b08361

[38] Y. Yao , K.‐T. Lee , X. Sheng , N. A. Batara , N. Hong , J. He , L. Xu , M. M. Hussain , H. A. Atwater , N. S. Lewis , R. G. Nuzzo , J. A. Rogers , Adv. Energy Mater. 2017, 7, 1601992.

[39] Y. Hou , Z. Wang , C. Cai , X. Hao , D. Li , N. Zhao , Y. Zhao , L. Chen , H. Ma , J. Xu , Adv. Mater. 2018, 30, 1704131. 10.1002/adma.201704131 29315825

[40] J. Hiller , J. D. Mendelsohn , M. F. Rubner , Nat. Mater. 2002, 1, 59. 12618851 10.1038/nmat719

[41] C. Ji , Z. Zhang , K. D. Omotosho , D. Berman , B. Lee , R. Divan , S. Guha , E. V. Shevchenko , ACS Nano 2022, 16, 14754. 36049118 10.1021/acsnano.2c05592

[42] W. Wu , Y. Xu , X. Ma , Z. Tian , C. Zhang , J. Han , X. Han , S. He , G. Duan , Y. Li , Adv. Funct. Mater. 2023, 33, 2302351.

[43] Y. Peng , L. Fan , W. Jin , Y. Ye , Z. Huang , S. Zhai , X. Luo , Y. Ma , J. Tang , J. Zhou , L. C. Greenburg , A. Majumdar , S. Fan , Y. Cui , Nat. Sustainability 2021, 5, 339.

