# Supplementary material for: Radiative Warming Glass for High‐Latitude Cold Regions
Source: Adv Sci (Weinh). 2025 Jan 10;12(9):2414192. doi: 10.1002/advs.202414192 (PMC11884607; doi:10.1002/advs.202414192)
Supplement: Supplementary file 1 — Supporting Information [file ADVS-12-2414192-s001.docx]

Supporting information

**Radiative Warming Glass for High-Latitude Cold Regions**

Zhengui Zhou^1,2^, Rong Liu^2^, Zhen Huang^1^, Bin Hu^1,3,*^ & Yi Long^2,*^

^1^Wuhan National Laboratory for Optoelectronics, School of Optical and Electronic Information, Huazhong University of Science and Technology, Wuhan Hubei 430074, P. R. China.

^2^Department of Electronic Engineering, The Chinese University of Hong Kong, Shatin, New Territories, Hong Kong SAR 999077, China.

^3^Shenzhen Huazhong University of Science and Technology Research Institute, Shenzhen 518057, China.

^*^Correspondence author: yilong@cuhk.edu.hk, [bin.hu@hust.edu.cn](mailto:bin.hu@hust.edu.cn).


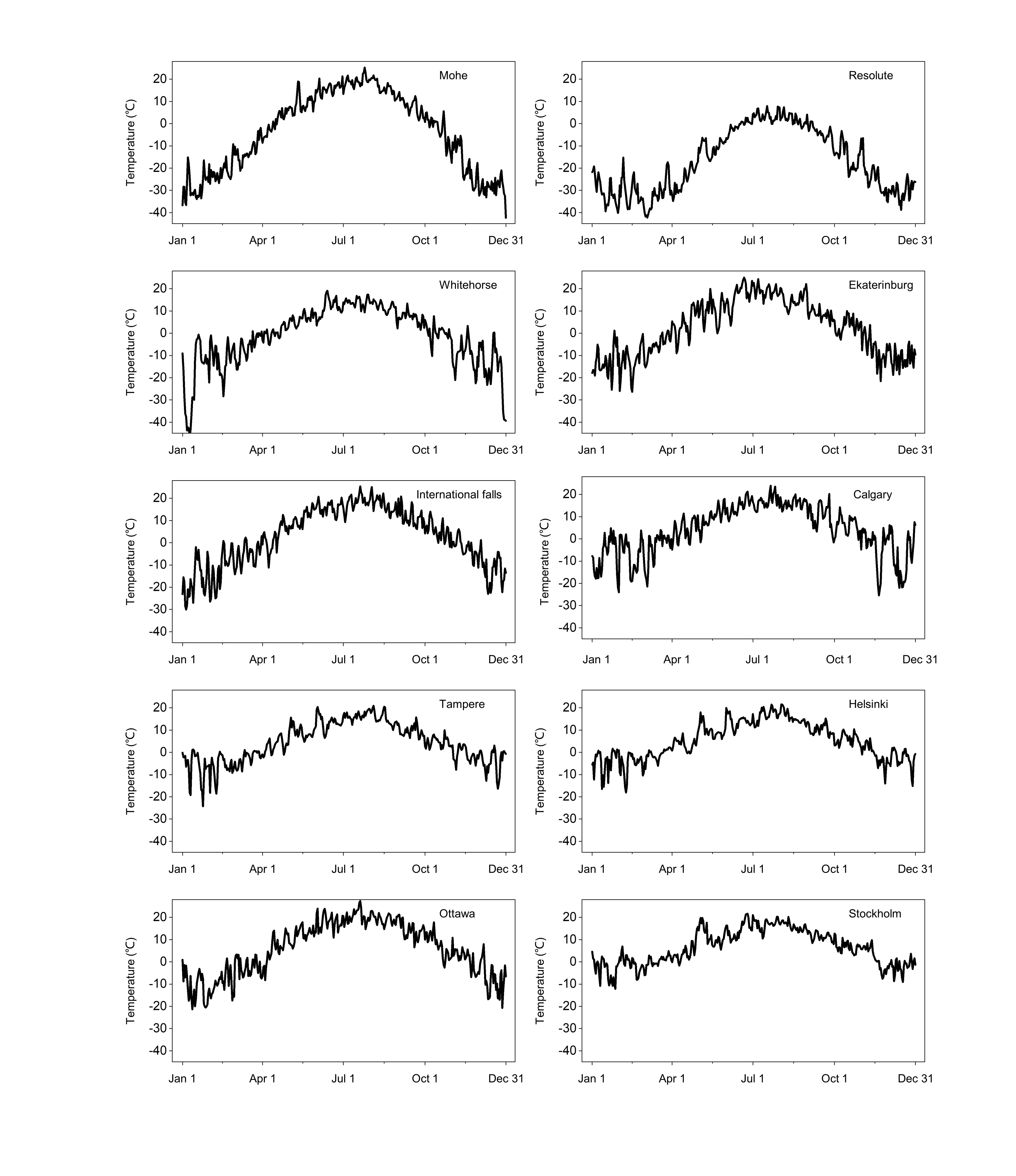


**Fig. S1.** The daily average temperatures throughout the year of 10 cities in high-latitude regions.


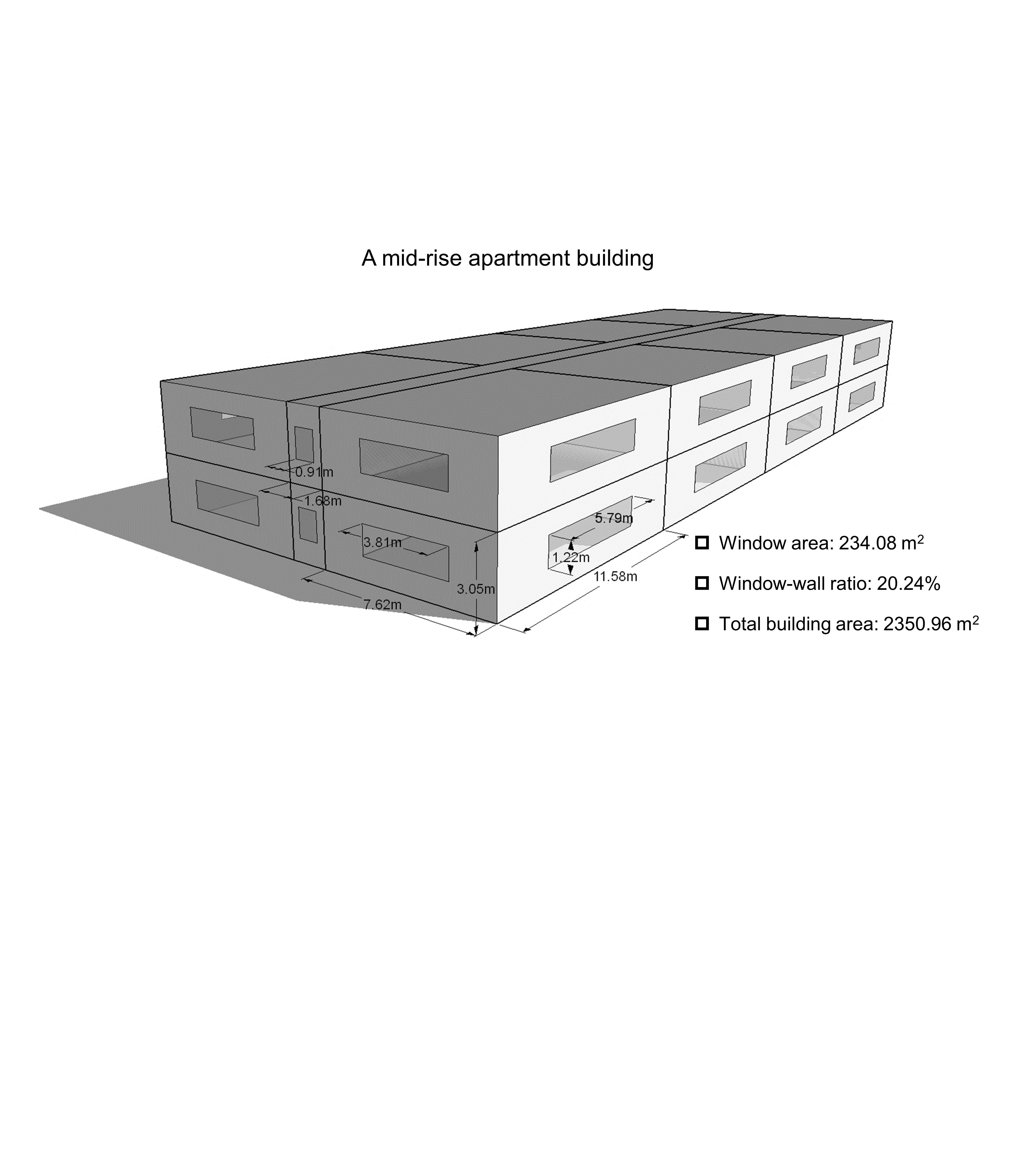


**Fig. S2.** The mid-rise apartment building used in the energy consumption simulation. The window area is 234.08 m^2^, the window-wall ratio is 20.24%, and the total building area is 2350.96 m^2^.


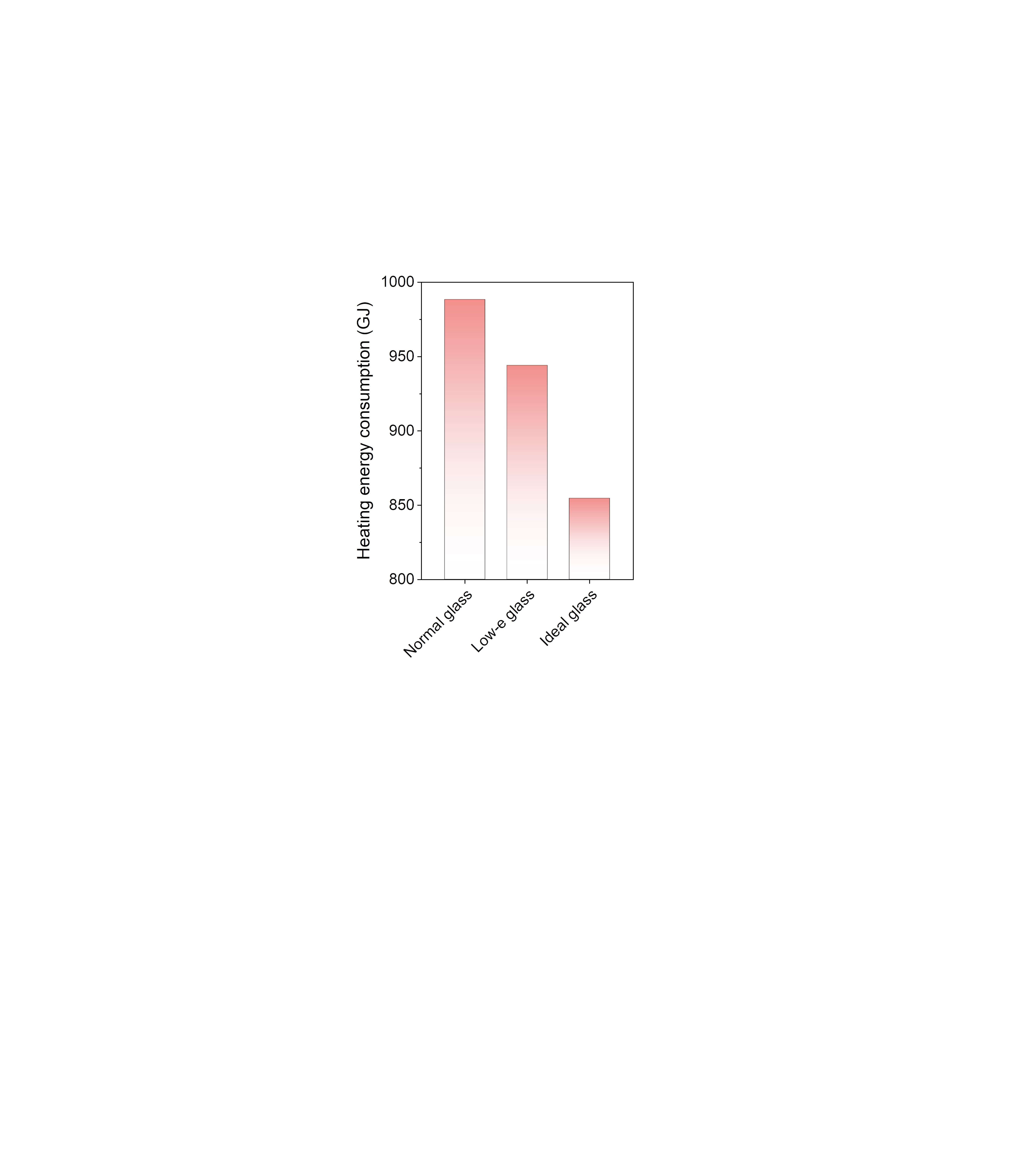


**Fig. S3.** Estimated heating energy consumption for a mid-rise apartment building using normal glass, commercial low-e glass, and ideal glass, respectively.

**
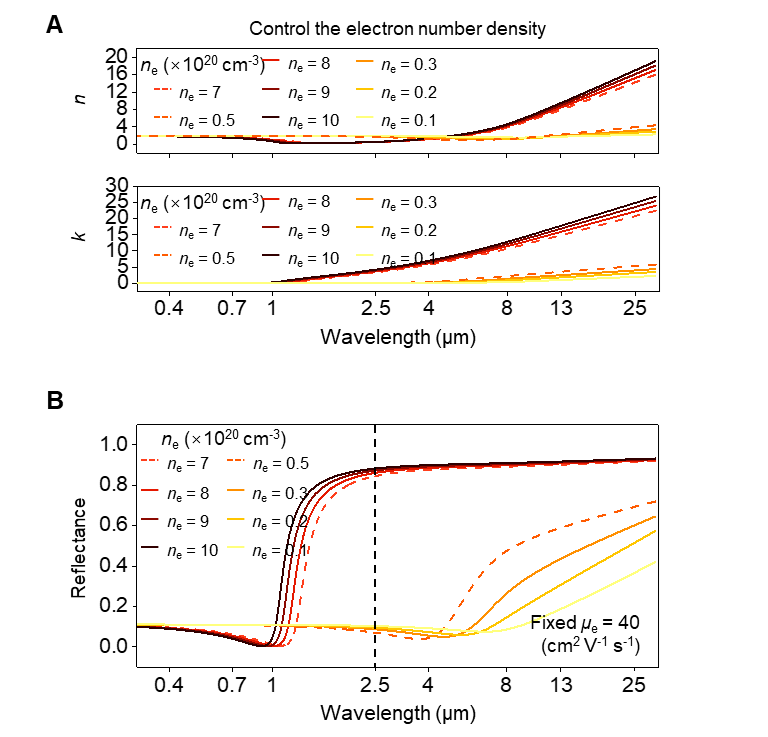
**

**Fig. S4.** (A) Calculated refractive index *n* and extinction coefficient *κ* at different *n*_e_ with fixing *μ*_e_. (B) Calculated reflectance spectra for varying *n*_e_ with fixing *μ*_e_.


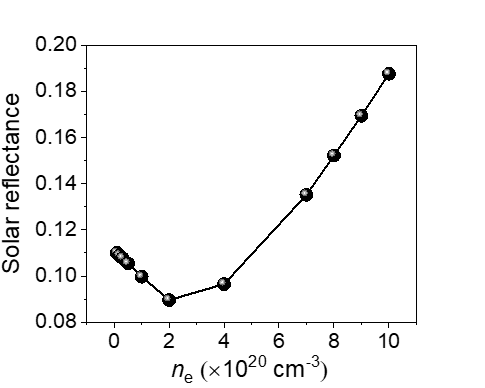


**Fig. S5.** Calculated solar reflectance for varying *n*_e_.


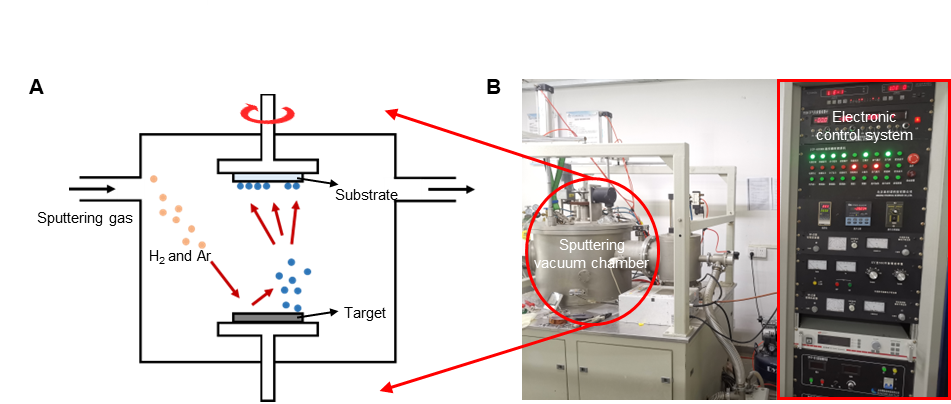


**Fig. S6.** (**A**) Schematic diagram illustrating the sputtering process in the magnetron sputtering chamber. (**B**) Image of the magnetron sputtering device.


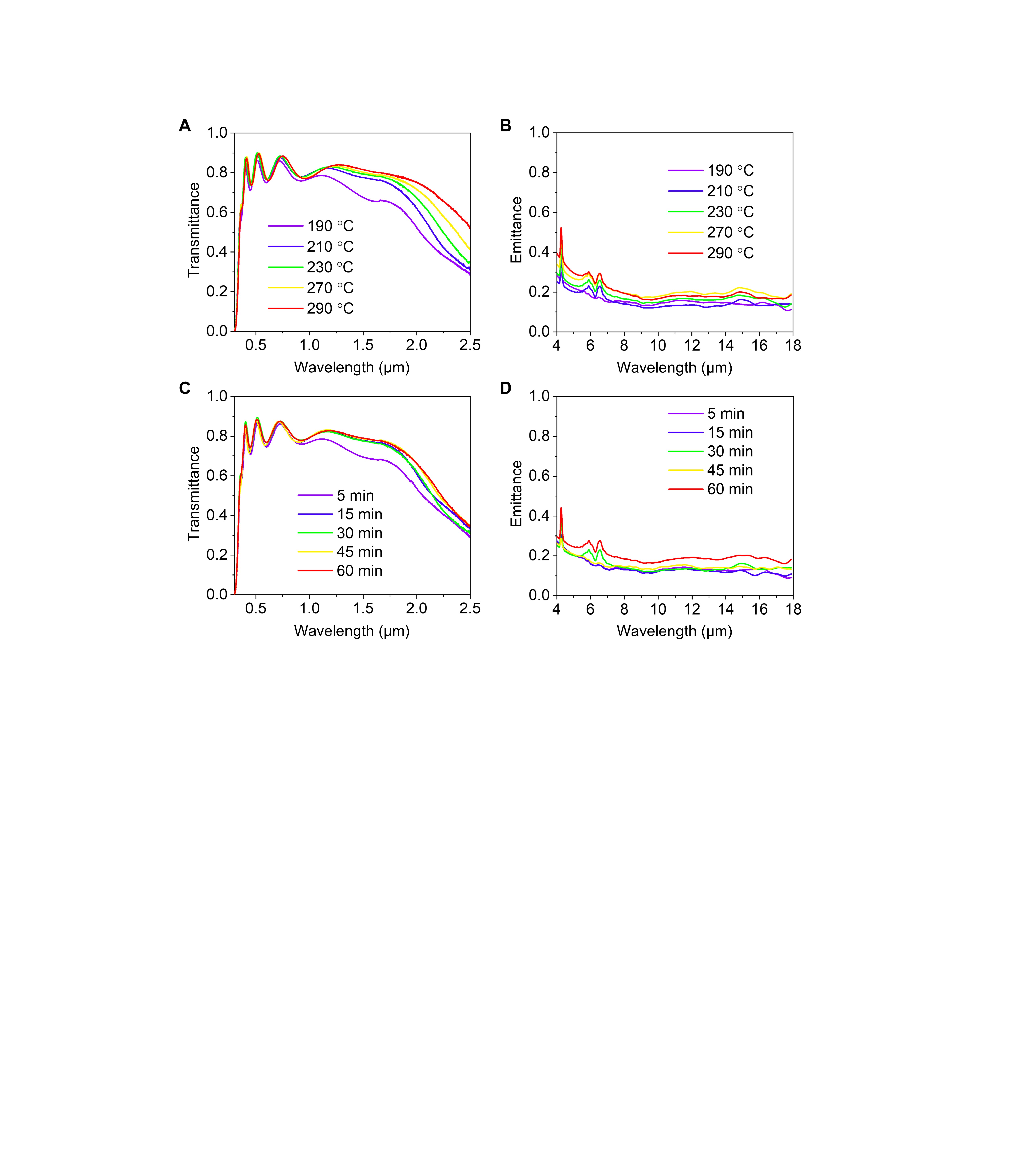


**Fig. S7.** (**A**) Transmittance of IHO glass under different post-annealing temperatures at fixing time of 30 min. (**B**) Emittance of IHO glass under different post-annealing temperatures at fixing time of 30 min. (**C**) Transmittance of IHO glass under different post-annealing time at fixing temperature of 210 ℃. (**D**) Emittance of IHO glass under different post-annealing time at fixing temperature of 210 ℃.


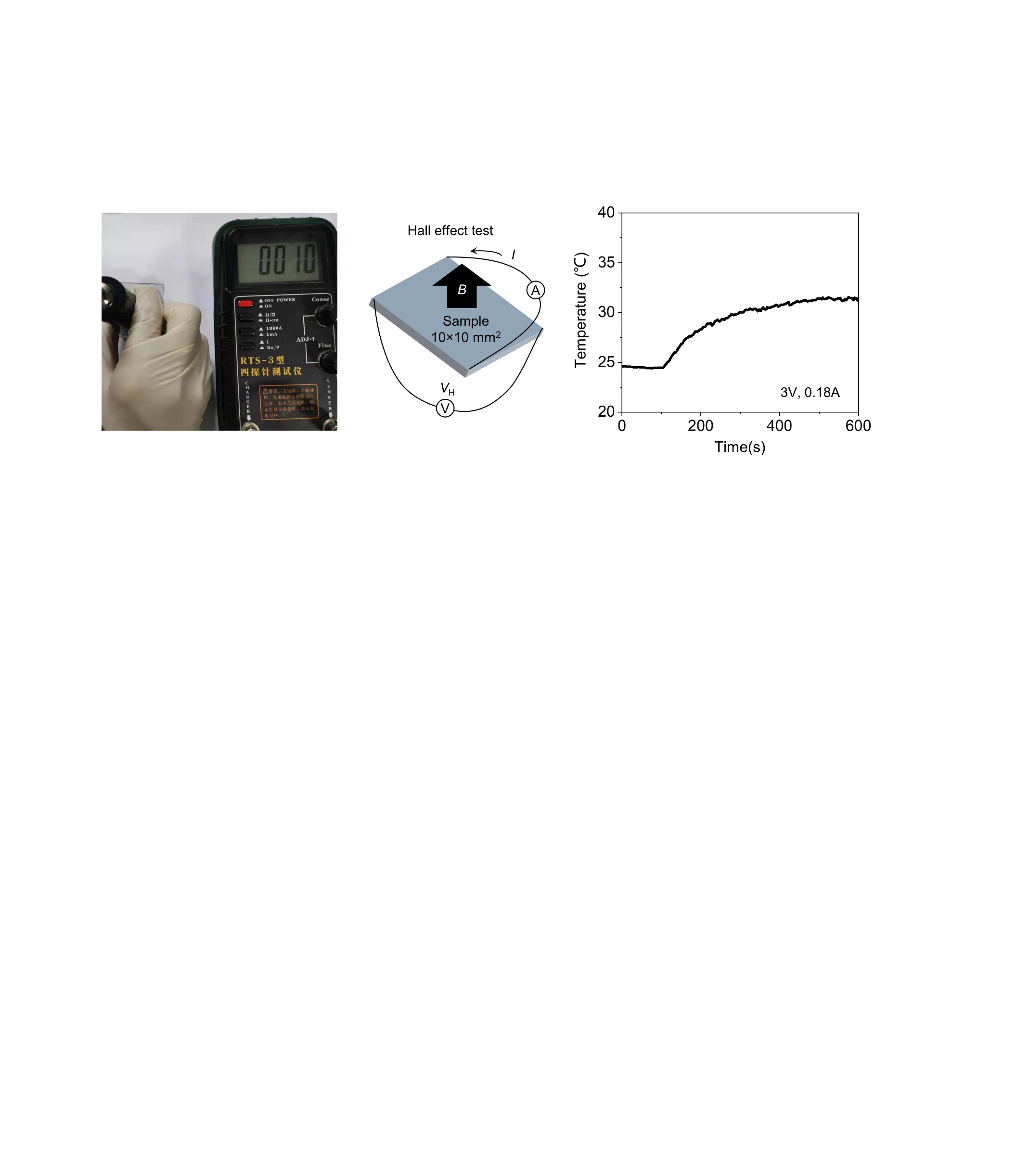


**Fig. S8.** Image of sheet resistance test for IHO glass. The result reveals a sheet resistance of 10 Ω/□.


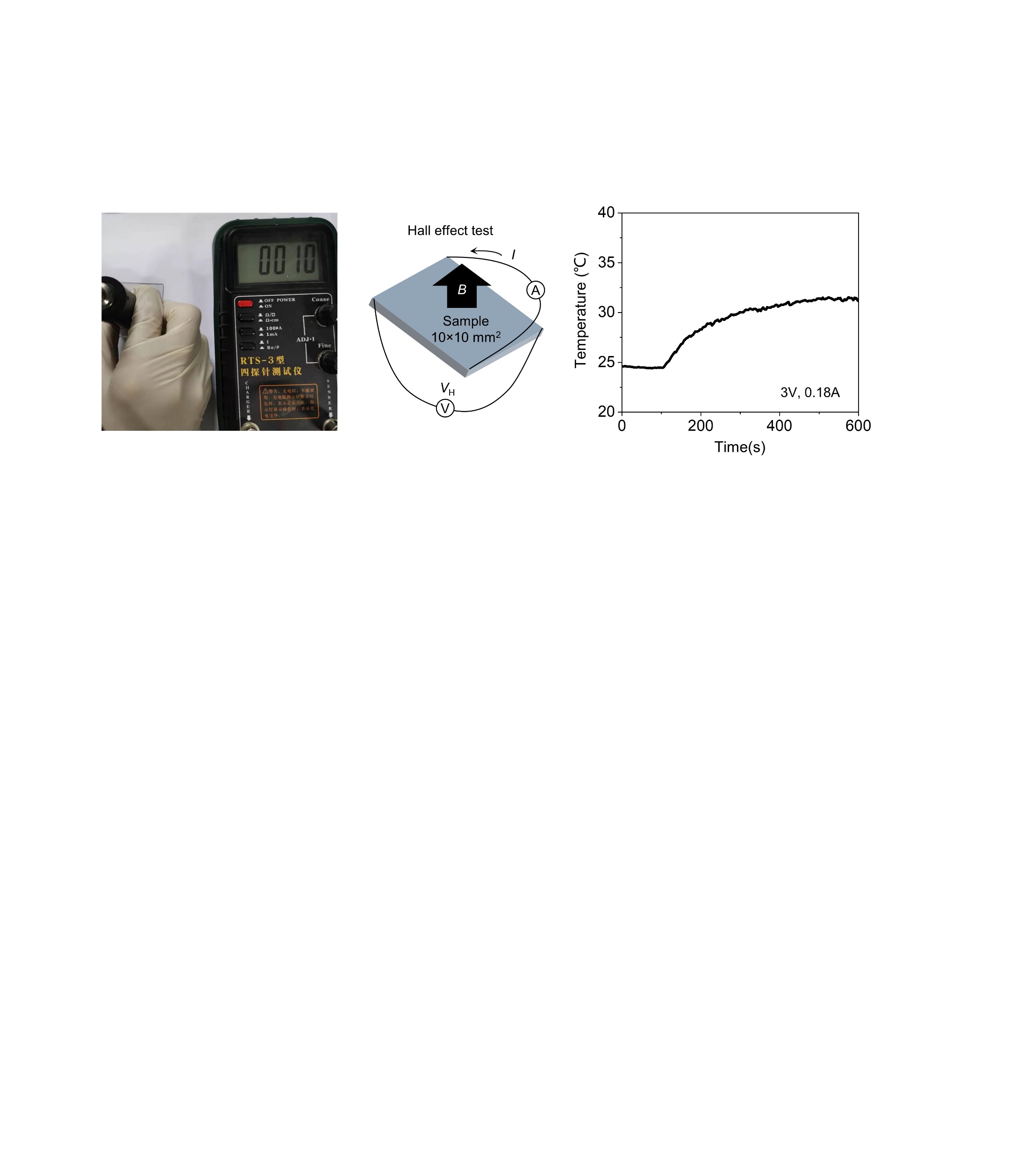


**Fig. S9.** Electric heating test of IHO glass. The applied voltage is 3V, and the current is 0.18A.


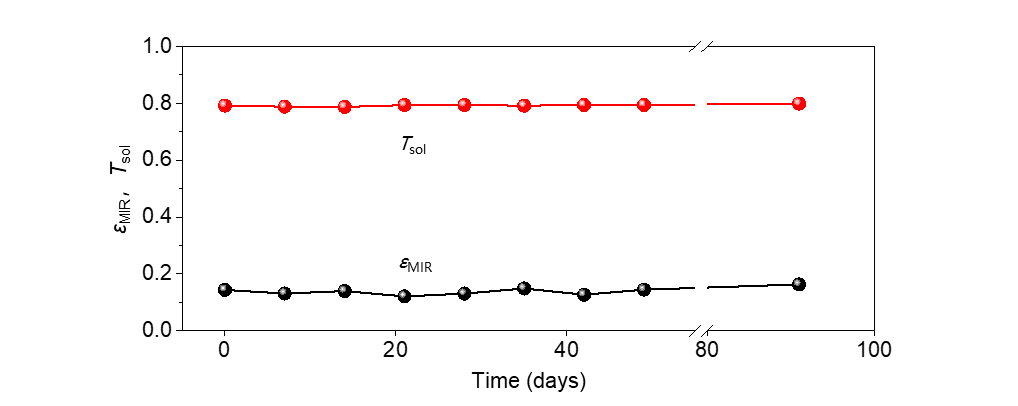


**Fig. S10.** The average *T*_sol_ and *ε*_MIR_ during 3 months of outdoor exposure. The overall experiment was conducted from Nov 24, 2021, to Feb 24, 2022, in Wuhan, China.


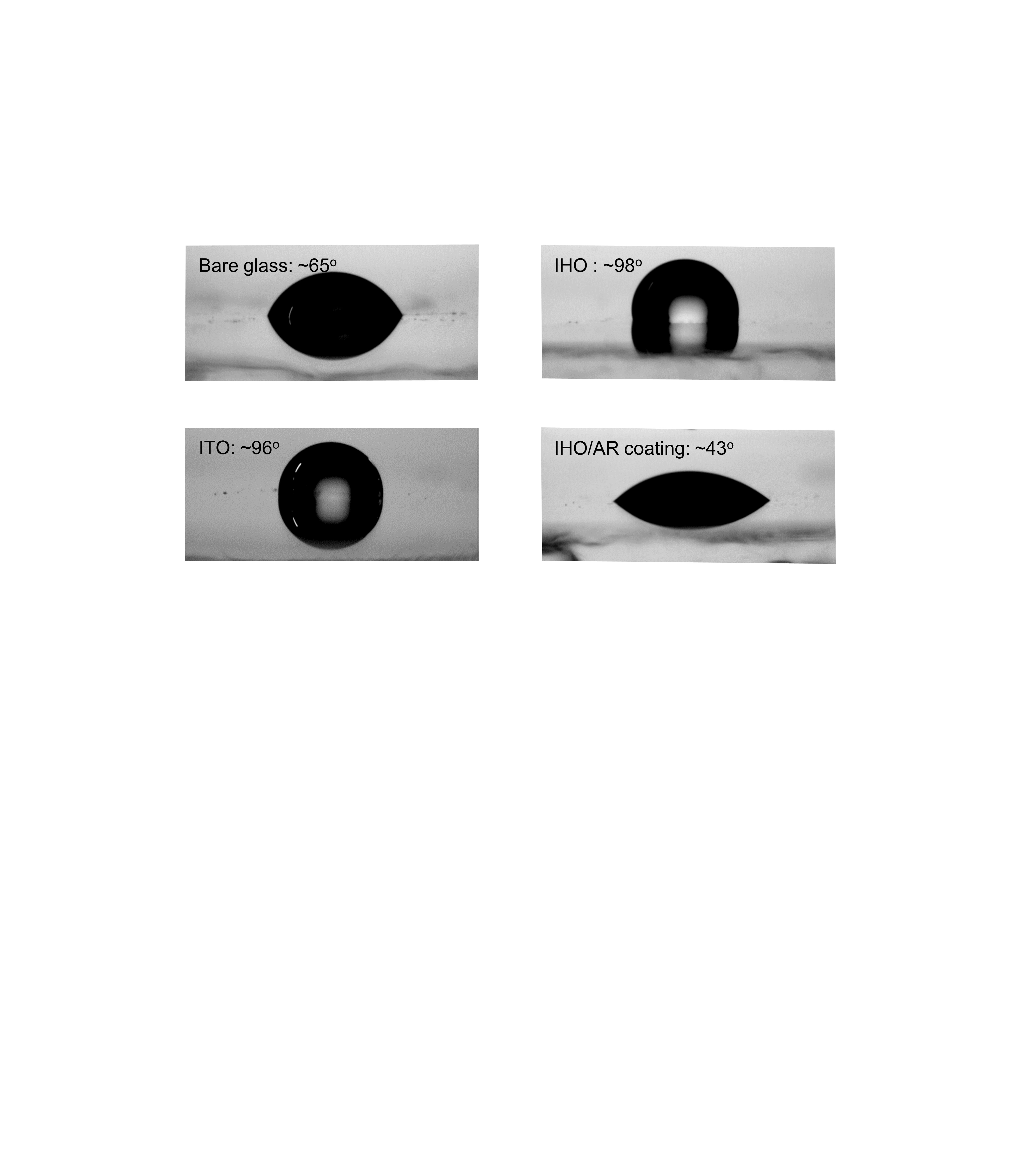


**Fig. S11.** Contact angle test results of different surfaces, including bare glass, ITO glass, IHO glass, and radiative warming glass (IHO/AR coating), showing corresponding contact angle of ~65°, ~96°, ~98°, and ~43°, respectively.


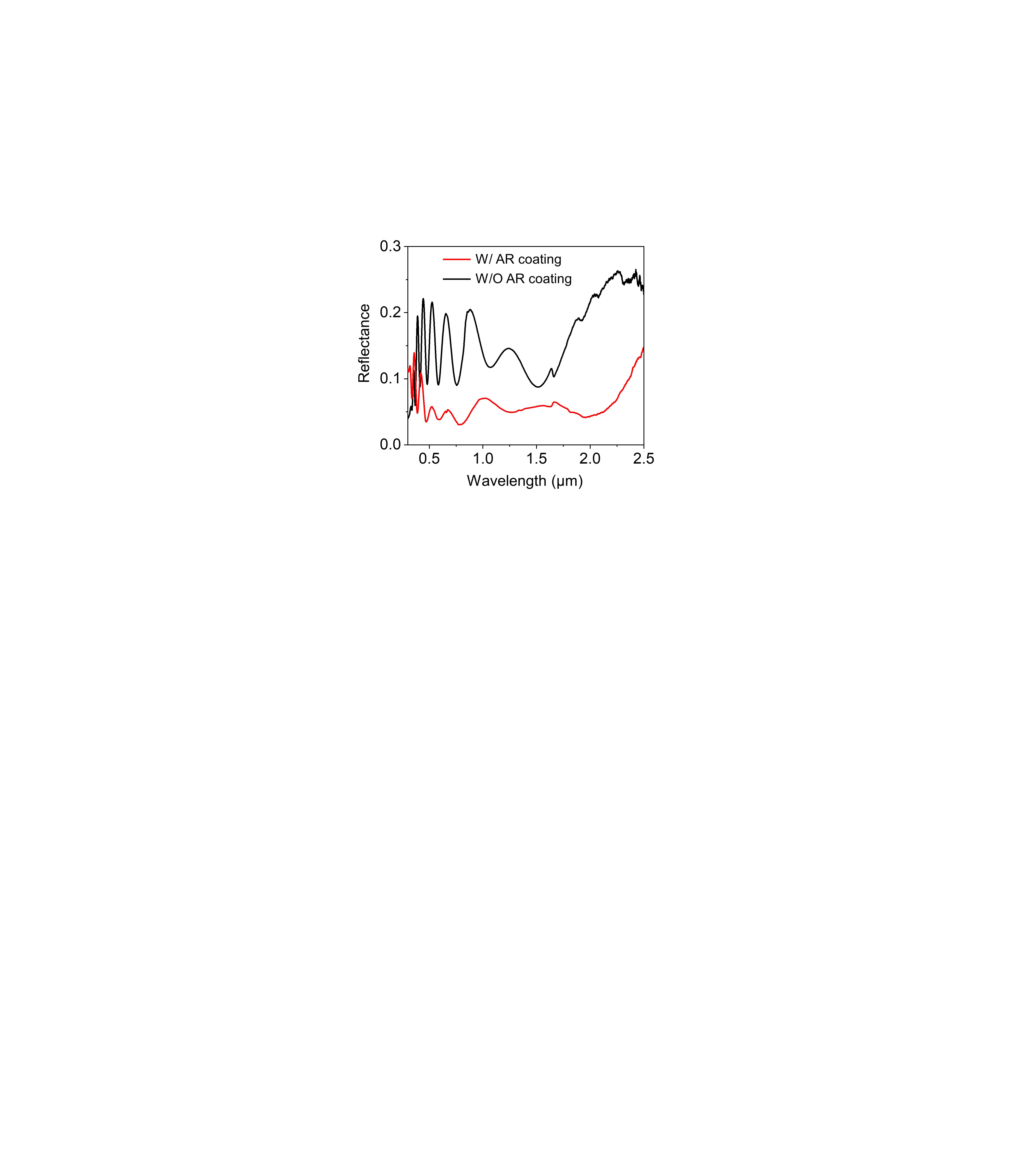


**Fig. S12.** Measured Reflectance of the IHO glass with and without SiO_2_ AR coating.


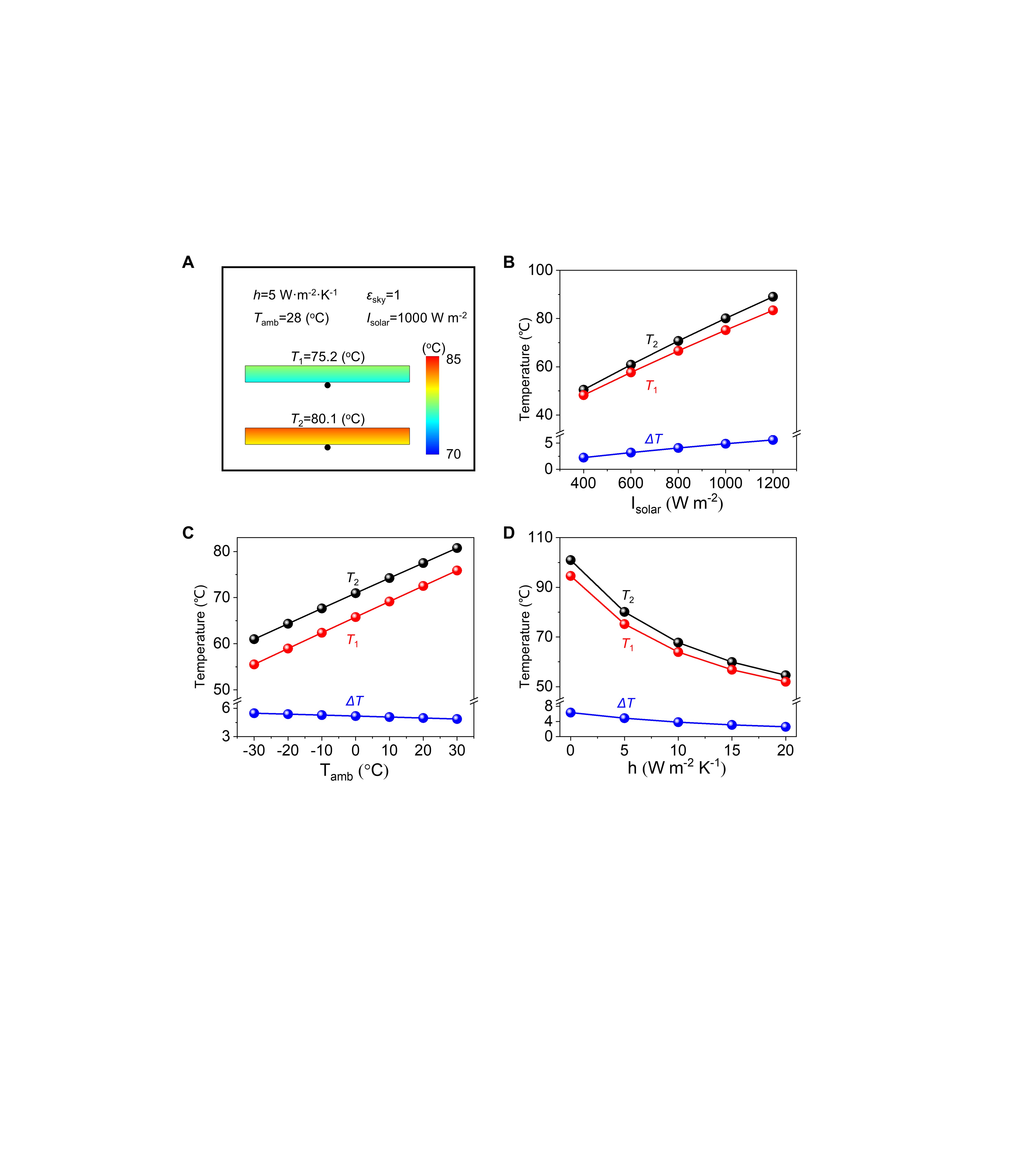


**Fig. S13.** (**A**) Simulated temperatures under a typical environmental condition. *T*_1_ and *T*_2_ denote the average temperatures of commercial ITO low-e glass and radiative warming glass, respectively. (**B**) Influence of solar irradiance (*I*_solar_) on temperature differences (Δ*T* = *T*_2_ - *T*_1_). (**C**) Influence of ambient temperature (*T*_amb_) on Δ*T*. (**D**) Influence of non-radiative heat transfer coefficient (*h*) on Δ*T*.


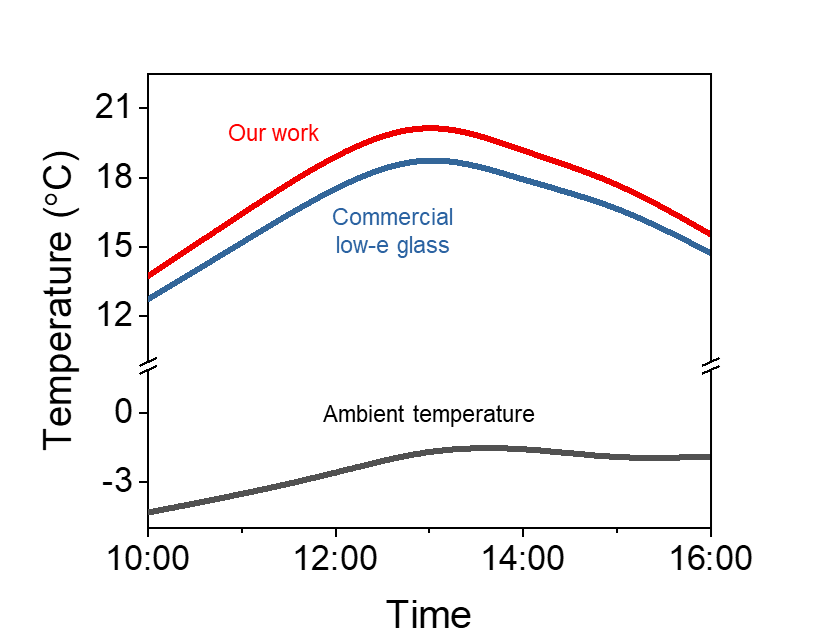


Fig. S14. Comparison of the simulated surface temperature of an eastern window using radiative warming glass versus commercial low-e glass from 10:00 to 16:00 for a typical apartment building in Ottawa.


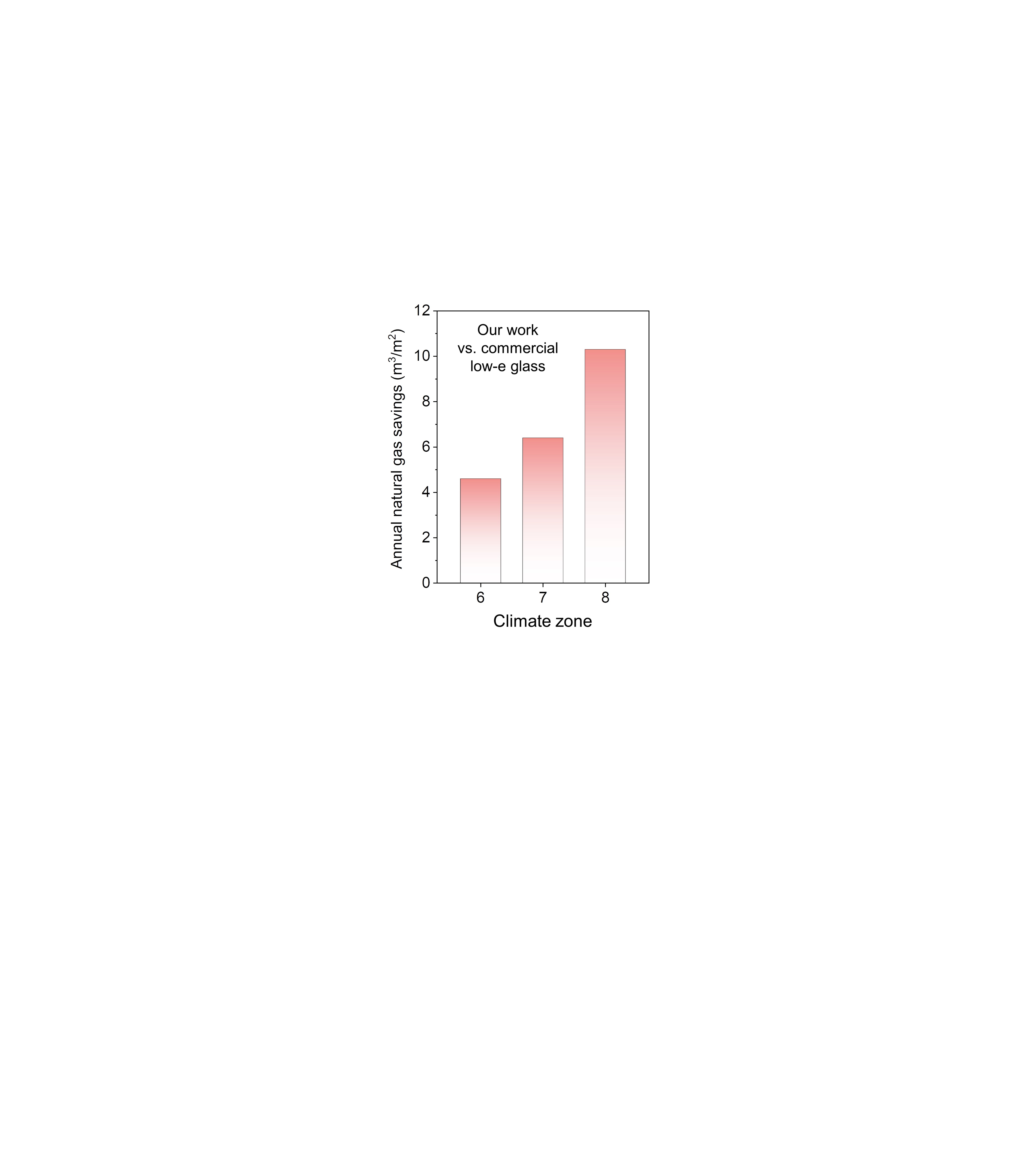


**Fig. S15.** Annually estimated natural gas savings in climate zones 6 to 8 globally, utilizing our radiative warming glass in comparison to commercial low-e glass.

**Supplemental Text**

Solid heat transfer simulation

The steady-state temperature simulations (Fig. S11) were conducted using the heat transfer module in COMSOL software, employing the surface-to-surface radiation physics interface ^[1-2]^. The geometric model was constructed with an external radiation source applied to its top surface. Our simulations assumed a typical ambient temperature (*T*_amb_) of 28°C, a sky emissivity (*ε*_sky_) of 1, and a non-radiative heat transfer coefficient of 5. The optical properties of the glass sample were modified according to the measured spectra. Notably, the calculated steady-state temperatures closely matched the experimental measurements. Furthermore, we explored the impact of environmental variables on temperature fluctuations by conducting simulations with varying factors such as solar intensity (*I*_solar_), non-radiative heat transfer coefficient (*h*), and ambient temperature (*T*_amb_).

Temperature simulation

A simulated temperature curve was developed using EnergyPlus based on a simple apartment with dimensions of 8 m in length, 6 m in width, and 2.7 m in height (Fig. S14). The total floor area of the apartment is 48 m^2^, and the total external wall surface area is 75.6 m^2^. To account for orientation effects, four windows were installed, each measuring 3 m in width and 2 m in height, covering 31.7% of the total wall surface area. The simulation utilized hourly weather data representative of a typical meteorological year, specifically employing weather data from Ottawa as the external boundary conditions.

Preliminary cost analysis

In this section, we outline the cost calculation process for the production of IHO glass.

1.Cost calculation of IHO glass without material loss:

The thickness of the IHO coating is defined as 370 nm (0.00037 mm), and the target material cost is approximately $110 per piece, covering an area of 0.01 m². The target volume is calculated as follows:

*V*_target,IHO_​=0.01 m^2^×0.001 m=0.00001 m^3^

The volume of IHO coating required for a coating area (*A*_glass_​) of 1 m² is:

*V*_coating,IHO_​=1 m^2^×0.00037 mm=0.00000037 m^3^

The number of target pieces required is:

*N*_IHO​_=*V*_coating,IHO_​/*V*_target,IHO_​​=0.00000037​/0.00001=0.037

Thus, the cost of producing IHO glass is approximately:

*C*_unit, IHO_​=*N*_IHO_​×Target Cost=0.037×110≈4.07 USD/m^2^

2.Cost calculation including the SiO_2_​ layer:

The thickness of the SiO_2_​ coating is 65 nm (0.000065 mm). The volume of SiO_2_​ required for an area of 1 m² is:

*V*_coating,SiO2​_=1 m^2^×0.000065 mm=0.000000065 m^3^

The number of target pieces needed for the SiO_2​_ coating is:

*N*_SiO2_​=*V*_coating,SiO2_​​/*V*_target,SiO2_​=0.000000065​/0.00001=0.0065

The cost of the SiO_2_​ coating is then:

*C*_SiO2​_=*N*_SiO2_​×Target Cost=0.0065×110≈0.72 USD/m^2^

3.Total cost of IHO glass including the SiO_2_​ layer:

Finally, the total cost of IHO glass, including the SiO_2_​ layer, is given by:

*C*_total​_=*C*_unit, IHO, actual_+*C*_SiO2​_=4.07+0.72≈4.79 USD/m^2^

In comparison, traditional low-e glass typically consists of multiple layers, including costly materials such as silver. The production costs for low-e glass can vary widely, ranging from $50 to $320 per square meter (Table S6), depending on the specific type and complexity of the coatings used.

**Supplemental Tables**

**Table S1. Selected papers on energy savings in various zones.**

| Climate zone | Ideal window spectrum | Reference |
| --- | --- | --- |
| 0-2:  Representative city (Singapore) | High *T*_vis_ = max  Low *T*_NIR_ = min  High *ε*_MIR_ = max | Joule 3, 3088 (2019)^[3]^;  Joule, doi.org/10.1016/j.joule.2024.07.020^[4]^;  Light: Sci. Appl. 11, 122 (2022)^[5]^;  Adv. Funct. Mater. 33, 2207940 (2023)^[6]^;  Science 382, 691 (2023)^[7]^;  Science 382, 684 (2023)^[8]^ |
| 3-5:  Representative city (New York) | Summer:  High *T*_vis_ = max  Low *T*_NIR_ = min  High *ε*_MIR_ = max | Science 374, 6574 (2021)^[9]^;  Sci. Adv. 8, 7359 (2022)^[10]^;  Nat. Sustain. 6, 428 (2023)^[11]^;  Nat. Commun. 14, 5087 (2023)^[12]^;  ACS Energy Lett. 6, 3906 (2021)^[13]^;  ACS Energy Lett. 7, 1758 (2022)^[14]^ |
|  | Winter:  High *T*_vis_ = max  High *T*_NIR_ = max  Low *ε*_MIR_ = min |  |
| 6-8:  Representative city (Stockholm) | High *T*_vis_ = max  High *T*_NIR_ = max  Low *ε*_MIR_ = min | This work;  Nano Energy 62, 111 (2019)^[15]^. (A novel silver nanowire mesh network was developed, but *T*_NIR_ was not provided) |

**Table S2.** **Summary of reported low-e glass.**

| Materials/Structure | *T*_vis_ | *T*_sol_ | *ε*_MIR_ | Reference |
| --- | --- | --- | --- | --- |
| SiN/Ag/SiN/glass | 0.746 | <0.5 | 0.03 | ^[16]^ |
| Al_2_O_3_/AlN/Ag/AlN/Ag/AlN/glass | 0.8 | <0.5 | 0.048 | ^[17]^ |
| AgNWs/PVB/glass | 0.83 | N/A | 0.302 | ^[15]^ |
| Aligned AgNWs/glass | 0.781 | N/A | 0.33 | ^[18]^ |
| AZO/glass | 0.818 | N/A | 0.41 | ^[19]^ |

*N/A: not available

**Table S3. A comparison between the radiative warming glass and commercial ITO low-e glass.**

| Optical properties | ITO low-e glass | Our work |
| --- | --- | --- |
| *T*_sol_ | 0.754 | 0.836 |
| *R*_sol_ | 0.145 | 0.054 |
| *T*_vis_ | 0.838 | 0.877 |
| *R*_vis_ | 0.113 | 0.053 |
| *T*_NIR_ | 0.702 | 0.822 |
| *R*_MIR_ | 0.854 | 0.883 |
| *ε*_MIR_ | 0.146 | 0.117 |
| *ξ* | 5.16 | 7.15 |

**Table S4. Summary of commercial low-e glass from the International Glazing Database.**

| **Number** | **ID** | ***T*_sol_** | ***T*_vis_** | ***ε*_MIR_** |
| --- | --- | --- | --- | --- |
| 1 | 11461 | 0.424 | 0.761 | 0.013 |
| 2 | 11460 | 0.436 | 0.768 | 0.013 |
| 3 | 20734 | 0.353 | 0.787 | 0.013 |
| 4 | 4474 | 0.305 | 0.673 | 0.014 |
| 5 | 4475 | 0.301 | 0.668 | 0.014 |
| 6 | 4476 | 0.297 | 0.663 | 0.014 |
| 7 | 21150 | 0.125 | 0.308 | 0.015 |
| 8 | 21153 | 0.133 | 0.306 | 0.015 |
| 9 | 21145 | 0.179 | 0.466 | 0.015 |
| 10 | 21141 | 0.248 | 0.57 | 0.015 |
| 11 | 21148 | 0.161 | 0.388 | 0.015 |
| 12 | 21138 | 0.238 | 0.559 | 0.015 |
| 13 | 21152 | 0.147 | 0.34 | 0.016 |
| 14 | 21135 | 0.246 | 0.569 | 0.016 |
| 15 | 21144 | 0.189 | 0.483 | 0.016 |
| 16 | 21140 | 0.249 | 0.572 | 0.016 |
| 17 | 21154 | 0.108 | 0.249 | 0.016 |
| 18 | 21142 | 0.247 | 0.569 | 0.016 |
| 19 | 21137 | 0.24 | 0.562 | 0.016 |
| 20 | 21146 | 0.162 | 0.435 | 0.016 |
| 21 | 21103 | 0.343 | 0.773 | 0.016 |
| 22 | 21149 | 0.147 | 0.359 | 0.016 |
| 23 | 21136 | 0.243 | 0.565 | 0.016 |
| 24 | 20913 | 0.234 | 0.516 | 0.016 |
| 25 | 21106 | 0.344 | 0.771 | 0.016 |
| 26 | 21101 | 0.334 | 0.762 | 0.016 |
| 27 | 21104 | 0.346 | 0.775 | 0.016 |
| 28 | 21100 | 0.338 | 0.767 | 0.016 |
| 29 | 21102 | 0.331 | 0.758 | 0.016 |
| 30 | 21105 | 0.344 | 0.773 | 0.016 |
| 31 | 20912 | 0.258 | 0.572 | 0.016 |
| 32 | 21078 | 0.172 | 0.509 | 0.016 |
| 33 | 14602 | 0.242 | 0.642 | 0.016 |
| 34 | 14600 | 0.245 | 0.646 | 0.016 |
| 35 | 14615 | 0.259 | 0.666 | 0.016 |
| 36 | 14618 | 0.258 | 0.663 | 0.016 |
| 37 | 14626 | 0.144 | 0.375 | 0.016 |
| 38 | 14640 | 0.158 | 0.47 | 0.016 |
| 39 | 14603 | 0.255 | 0.662 | 0.016 |
| 40 | 14616 | 0.258 | 0.665 | 0.016 |
| 41 | 14628 | 0.119 | 0.309 | 0.016 |
| 42 | 14630 | 0.079 | 0.198 | 0.016 |
| 43 | 14638 | 0.1 | 0.252 | 0.016 |
| 44 | 14648 | 0.173 | 0.504 | 0.016 |
| 45 | 14606 | 0.25 | 0.655 | 0.016 |
| 46 | 14620 | 0.098 | 0.256 | 0.016 |
| 47 | 14604 | 0.254 | 0.659 | 0.016 |
| 48 | 14610 | 0.257 | 0.662 | 0.016 |
| 49 | 14612 | 0.256 | 0.661 | 0.016 |
| 50 | 14608 | 0.248 | 0.65 | 0.016 |
| 51 | 14613 | 0.259 | 0.667 | 0.016 |
| 52 | 14614 | 0.259 | 0.666 | 0.016 |
| 53 | 14646 | 0.19 | 0.54 | 0.016 |
| 54 | 14605 | 0.252 | 0.657 | 0.016 |
| 55 | 14636 | 0.127 | 0.321 | 0.016 |
| 56 | 21081 | 0.177 | 0.519 | 0.016 |
| 57 | 21076 | 0.175 | 0.516 | 0.016 |
| 58 | 20918 | 0.185 | 0.422 | 0.016 |
| 59 | 21080 | 0.178 | 0.521 | 0.016 |
| 60 | 21082 | 0.176 | 0.518 | 0.017 |
| 61 | 21077 | 0.174 | 0.512 | 0.017 |
| 62 | 20919 | 0.168 | 0.38 | 0.017 |
| 63 | 16671 | 0.22 | 0.468 | 0.017 |
| 64 | 20881 | 0.214 | 0.532 | 0.017 |
| 65 | 20880 | 0.216 | 0.536 | 0.017 |
| 66 | 20882 | 0.212 | 0.529 | 0.017 |
| 67 | 20884 | 0.22 | 0.541 | 0.017 |
| 68 | 20886 | 0.218 | 0.538 | 0.017 |
| 69 | 20885 | 0.219 | 0.54 | 0.018 |
| 70 | 20746 | 0.367 | 0.804 | 0.018 |
| 71 | 20744 | 0.369 | 0.807 | 0.018 |
| 72 | 20743 | 0.349 | 0.781 | 0.018 |
| 73 | 20742 | 0.353 | 0.787 | 0.018 |
| 74 | 20741 | 0.358 | 0.793 | 0.018 |
| 75 | 20745 | 0.368 | 0.805 | 0.018 |
| 76 | 20962 | 0.3 | 0.769 | 0.018 |
| 77 | 20963 | 0.297 | 0.764 | 0.018 |
| 78 | 21058 | 0.298 | 0.772 | 0.018 |
| 79 | 20961 | 0.303 | 0.774 | 0.018 |
| 80 | 21067 | 0.297 | 0.771 | 0.018 |
| 81 | 21054 | 0.292 | 0.764 | 0.018 |
| 82 | 20966 | 0.305 | 0.777 | 0.018 |
| 83 | 21056 | 0.3 | 0.777 | 0.018 |
| 84 | 21053 | 0.295 | 0.769 | 0.018 |
| 85 | 20965 | 0.307 | 0.78 | 0.019 |
| 86 | 21055 | 0.29 | 0.759 | 0.019 |
| 87 | 21057 | 0.299 | 0.774 | 0.019 |
| 88 | 20967 | 0.304 | 0.775 | 0.019 |
| 89 | 1344 | 0.194 | 0.453 | 0.019 |
| 90 | 20906 | 0.376 | 0.824 | 0.019 |
| 91 | 20904 | 0.38 | 0.828 | 0.019 |
| 92 | 4468 | 0.246 | 0.562 | 0.019 |
| 93 | 4470 | 0.24 | 0.553 | 0.019 |
| 94 | 4469 | 0.243 | 0.557 | 0.019 |
| 95 | 20898 | 0.362 | 0.809 | 0.019 |
| 96 | 19765 | 0.115 | 0.229 | 0.019 |
| 97 | 20897 | 0.366 | 0.814 | 0.019 |
| 98 | 20896 | 0.371 | 0.82 | 0.019 |
| 99 | 1334 | 0.163 | 0.344 | 0.019 |
| 100 | 21107 | 0.317 | 0.671 | 0.019 |
| 101 | 21108 | 0.312 | 0.667 | 0.019 |
| 102 | 28600 | 0.196 | 0.44 | 0.02 |
| 103 | 21109 | 0.308 | 0.662 | 0.02 |
| 104 | 21115 | 0.32 | 0.674 | 0.02 |
| 105 | 21110 | 0.304 | 0.658 | 0.02 |
| 106 | 21116 | 0.319 | 0.672 | 0.02 |
| 107 | 28601 | 0.251 | 0.543 | 0.02 |
| 108 | 20905 | 0.378 | 0.826 | 0.02 |
| 109 | 19766 | 0.174 | 0.338 | 0.02 |
| 110 | 4464 | 0.181 | 0.436 | 0.02 |
| 111 | 4460 | 0.183 | 0.439 | 0.02 |
| 112 | 4461 | 0.181 | 0.436 | 0.02 |
| 113 | 4459 | 0.186 | 0.443 | 0.02 |
| 114 | 4463 | 0.183 | 0.439 | 0.02 |
| 115 | 4462 | 0.186 | 0.443 | 0.02 |
| 116 | 20972 | 0.251 | 0.661 | 0.021 |
| 117 | 20975 | 0.26 | 0.677 | 0.021 |
| 118 | 20984 | 0.255 | 0.678 | 0.021 |
| 119 | 16866 | 0.419 | 0.745 | 0.021 |
| 120 | 16666 | 0.289 | 0.6 | 0.021 |
| 121 | 20977 | 0.258 | 0.673 | 0.021 |
| 122 | 20974 | 0.261 | 0.678 | 0.021 |
| 123 | 20969 | 0.258 | 0.674 | 0.021 |
| 124 | 20986 | 0.253 | 0.674 | 0.021 |
| 125 | 20893 | 0.372 | 0.814 | 0.021 |
| 126 | 20976 | 0.259 | 0.675 | 0.021 |
| 127 | 20981 | 0.248 | 0.665 | 0.021 |
| 128 | 4159 | 0.364 | 0.784 | 0.021 |
| 129 | 20970 | 0.255 | 0.67 | 0.021 |
| 130 | 20985 | 0.254 | 0.676 | 0.021 |
| 131 | 21065 | 0.253 | 0.661 | 0.021 |
| 132 | 21059 | 0.252 | 0.66 | 0.021 |
| 133 | 21061 | 0.246 | 0.652 | 0.021 |
| 134 | 20982 | 0.246 | 0.661 | 0.021 |
| 135 | 20980 | 0.25 | 0.669 | 0.021 |
| 136 | 21063 | 0.253 | 0.662 | 0.021 |
| 137 | 21060 | 0.25 | 0.656 | 0.021 |
| 138 | 21064 | 0.254 | 0.663 | 0.021 |
| 139 | 21062 | 0.244 | 0.648 | 0.021 |
| 140 | 21066 | 0.252 | 0.659 | 0.021 |
| 141 | 14617 | 0.252 | 0.658 | 0.021 |
| 142 | 20971 | 0.253 | 0.665 | 0.022 |
| 143 | 16668 | 0.263 | 0.511 | 0.022 |
| 144 | 20987 | 0.253 | 0.672 | 0.022 |
| 145 | 1335 | 0.159 | 0.341 | 0.022 |
| 146 | 20901 | 0.384 | 0.826 | 0.022 |
| 147 | 4448 | 0.506 | 0.84 | 0.022 |
| 148 | 4447 | 0.516 | 0.848 | 0.022 |
| 149 | 20875 | 0.168 | 0.406 | 0.022 |
| 150 | 20892 | 0.377 | 0.82 | 0.022 |
| 151 | 20902 | 0.382 | 0.824 | 0.022 |
| 152 | 21411 | 0.489 | 0.778 | 0.022 |
| 153 | 20878 | 0.368 | 0.656 | 0.022 |
| 154 | 20874 | 0.172 | 0.409 | 0.022 |
| 155 | 21409 | 0.499 | 0.782 | 0.022 |
| 156 | 20876 | 0.378 | 0.662 | 0.022 |
| 157 | 21408 | 0.416 | 0.715 | 0.022 |
| 158 | 20900 | 0.386 | 0.828 | 0.022 |
| 159 | 20877 | 0.375 | 0.66 | 0.022 |
| 160 | 21420 | 0.471 | 0.768 | 0.022 |
| 161 | 21410 | 0.499 | 0.783 | 0.022 |
| 162 | 21414 | 0.478 | 0.783 | 0.022 |
| 163 | 4483 | 0.245 | 0.559 | 0.022 |
| 164 | 21412 | 0.48 | 0.773 | 0.022 |
| 165 | 4485 | 0.238 | 0.55 | 0.022 |
| 166 | 21416 | 0.46 | 0.773 | 0.022 |
| 167 | 4484 | 0.241 | 0.555 | 0.022 |
| 168 | 21407 | 0.419 | 0.717 | 0.022 |
| 169 | 21415 | 0.469 | 0.778 | 0.022 |
| 170 | 21413 | 0.482 | 0.785 | 0.022 |
| 171 | 21417 | 0.452 | 0.768 | 0.022 |
| 172 | 20979 | 0.252 | 0.673 | 0.023 |
| 173 | 20879 | 0.372 | 0.658 | 0.023 |
| 174 | 20894 | 0.367 | 0.809 | 0.023 |
| 175 | 4158 | 0.248 | 0.514 | 0.023 |
| 176 | 20873 | 0.175 | 0.413 | 0.023 |
| 177 | 20788 | 0.154 | 0.34 | 0.023 |
| 178 | 20762 | 0.554 | 0.861 | 0.023 |
| 179 | 4157 | 0.264 | 0.523 | 0.023 |
| 180 | 14656 | 0.111 | 0.384 | 0.023 |
| 181 | 14652 | 0.108 | 0.376 | 0.023 |
| 182 | 14650 | 0.109 | 0.379 | 0.023 |
| 183 | 14658 | 0.11 | 0.381 | 0.023 |
| 184 | 1345 | 0.186 | 0.443 | 0.023 |
| 185 | 20787 | 0.188 | 0.419 | 0.023 |
| 186 | 20763 | 0.548 | 0.858 | 0.023 |
| 187 | 4156 | 0.213 | 0.422 | 0.023 |
| 188 | 20785 | 0.173 | 0.414 | 0.024 |
| 189 | 20764 | 0.543 | 0.855 | 0.024 |
| 190 | 21131 | 0.257 | 0.563 | 0.024 |
| 191 | 20791 | 0.346 | 0.769 | 0.024 |
| 192 | 20790 | 0.349 | 0.772 | 0.024 |
| 193 | 16865 | 0.428 | 0.75 | 0.024 |
| 194 | 20793 | 0.339 | 0.762 | 0.024 |
| 195 | 21095 | 0.346 | 0.772 | 0.024 |
| 196 | 21097 | 0.338 | 0.763 | 0.024 |
| 197 | 20792 | 0.343 | 0.765 | 0.024 |
| 198 | 21133 | 0.244 | 0.562 | 0.024 |
| 199 | 21096 | 0.342 | 0.767 | 0.024 |
| 200 | 21099 | 0.349 | 0.775 | 0.024 |
| 201 | 21098 | 0.334 | 0.758 | 0.024 |
| 202 | 21139 | 0.253 | 0.572 | 0.024 |
| 203 | 21134 | 0.241 | 0.559 | 0.024 |
| 204 | 21132 | 0.254 | 0.559 | 0.024 |
| 205 | 21225 | 0.361 | 0.654 | 0.024 |
| 206 | 21224 | 0.368 | 0.659 | 0.024 |
| 207 | 21226 | 0.355 | 0.65 | 0.024 |
| 208 | 28608 | 0.169 | 0.393 | 0.025 |
| 209 | 16662 | 0.35 | 0.748 | 0.025 |
| 210 | 20956 | 0.315 | 0.684 | 0.025 |
| 211 | 4151 | 0.365 | 0.761 | 0.025 |
| 212 | 4148 | 0.517 | 0.822 | 0.025 |
| 213 | 25562 | 0.245 | 0.557 | 0.025 |
| 214 | 4586 | 0.335 | 0.643 | 0.025 |
| 215 | 4584 | 0.353 | 0.658 | 0.025 |
| 216 | 4585 | 0.344 | 0.65 | 0.025 |
| 217 | 4150 | 0.349 | 0.745 | 0.025 |
| 218 | 4155 | 0.166 | 0.333 | 0.025 |
| 219 | 20784 | 0.204 | 0.484 | 0.025 |
| 220 | 4467 | 0.24 | 0.553 | 0.025 |
| 221 | 4466 | 0.243 | 0.557 | 0.025 |
| 222 | 4465 | 0.246 | 0.562 | 0.025 |
| 223 | 20955 | 0.321 | 0.69 | 0.025 |
| 224 | 25563 | 0.241 | 0.549 | 0.025 |
| 225 | 4471 | 0.305 | 0.673 | 0.025 |
| 226 | 4472 | 0.301 | 0.668 | 0.025 |
| 227 | 4473 | 0.297 | 0.663 | 0.025 |
| 228 | 14516 | 0.184 | 0.441 | 0.025 |
| 229 | 14472 | 0.17 | 0.426 | 0.025 |
| 230 | 14514 | 0.184 | 0.442 | 0.025 |
| 231 | 14512 | 0.182 | 0.438 | 0.025 |
| 232 | 14476 | 0.177 | 0.435 | 0.025 |
| 233 | 14478 | 0.174 | 0.432 | 0.025 |
| 234 | 14510 | 0.183 | 0.439 | 0.025 |
| 235 | 14518 | 0.183 | 0.44 | 0.025 |
| 236 | 14517 | 0.181 | 0.437 | 0.025 |
| 237 | 14470 | 0.172 | 0.429 | 0.025 |
| 238 | 16860 | 0.438 | 0.757 | 0.026 |
| 239 | 11137 | 0.255 | 0.565 | 0.026 |
| 240 | 11136 | 0.257 | 0.566 | 0.026 |
| 241 | 11132 | 0.258 | 0.568 | 0.026 |
| 242 | 21073 | 0.255 | 0.673 | 0.026 |
| 243 | 21069 | 0.249 | 0.663 | 0.026 |
| 244 | 21072 | 0.256 | 0.675 | 0.026 |
| 245 | 21068 | 0.251 | 0.667 | 0.026 |
| 246 | 21074 | 0.254 | 0.671 | 0.026 |
| 247 | 21070 | 0.246 | 0.658 | 0.026 |
| 248 | 28607 | 0.172 | 0.397 | 0.026 |
| 249 | 21074 | 0.254 | 0.671 | 0.026 |
| 250 | 21070 | 0.246 | 0.658 | 0.026 |
| 251 | 28607 | 0.172 | 0.397 | 0.026 |
| 252 | 11593 | 0.467 | 0.812 | 0.026 |
| 253 | 21405 | 0.35 | 0.789 | 0.026 |
| 254 | 21119 | 0.313 | 0.67 | 0.026 |
| 255 | 21404 | 0.352 | 0.792 | 0.027 |
| 256 | 28024 | 0.252 | 0.539 | 0.027 |
| 257 | 28023 | 0.272 | 0.559 | 0.027 |
| 258 | 28025 | 0.258 | 0.545 | 0.027 |
| 259 | 28026 | 0.246 | 0.533 | 0.027 |
| 260 | 21406 | 0.345 | 0.784 | 0.027 |
| 261 | 21111 | 0.312 | 0.671 | 0.027 |
| 262 | 21126 | 0.154 | 0.36 | 0.027 |
| 263 | 21122 | 0.2 | 0.511 | 0.027 |
| 264 | 21118 | 0.314 | 0.672 | 0.027 |
| 265 | 21112 | 0.308 | 0.667 | 0.027 |
| 266 | 4152 | 0.4 | 0.812 | 0.027 |
| 267 | 13121 | 0.446 | 0.756 | 0.027 |
| 268 | 13130 | 0.426 | 0.749 | 0.027 |
| 269 | 21129 | 0.171 | 0.362 | 0.027 |
| 270 | 20916 | 0.212 | 0.448 | 0.027 |
| 271 | 21124 | 0.199 | 0.455 | 0.027 |
| 272 | 21120 | 0.236 | 0.568 | 0.027 |
| 273 | 14494 | 0.194 | 0.442 | 0.027 |
| 274 | 14480 | 0.181 | 0.429 | 0.027 |
| 275 | 14482 | 0.178 | 0.426 | 0.027 |
| 276 | 14486 | 0.186 | 0.435 | 0.027 |
| 277 | 14488 | 0.183 | 0.432 | 0.027 |
| 278 | 14492 | 0.192 | 0.438 | 0.027 |
| 279 | 14496 | 0.193 | 0.441 | 0.027 |
| 280 | 14497 | 0.191 | 0.437 | 0.027 |
| 281 | 14498 | 0.193 | 0.44 | 0.027 |
| 282 | 14490 | 0.192 | 0.439 | 0.027 |
| 283 | 11592 | 0.477 | 0.817 | 0.027 |
| 284 | 21128 | 0.19 | 0.402 | 0.027 |
| 285 | 21117 | 0.316 | 0.674 | 0.027 |
| 286 | 21113 | 0.304 | 0.662 | 0.027 |
| 287 | 21130 | 0.139 | 0.294 | 0.027 |
| 288 | 21114 | 0.301 | 0.658 | 0.027 |
| 289 | 20915 | 0.235 | 0.496 | 0.027 |
| 290 | 21125 | 0.183 | 0.421 | 0.027 |
| 291 | 21121 | 0.222 | 0.548 | 0.027 |
| 292 | 11591 | 0.482 | 0.82 | 0.027 |
| 293 | 20921 | 0.163 | 0.335 | 0.027 |
| 294 | 4499 | 0.338 | 0.685 | 0.027 |
| 295 | 21094 | 0.391 | 0.762 | 0.027 |
| 296 | 11595 | 0.45 | 0.803 | 0.027 |
| 297 | 20922 | 0.147 | 0.302 | 0.027 |
| 298 | 21089 | 0.379 | 0.753 | 0.027 |
| 299 | 11584 | 0.485 | 0.825 | 0.027 |
| 300 | 21092 | 0.394 | 0.765 | 0.027 |
| 301 | 11594 | 0.458 | 0.808 | 0.027 |
| 302 | 11590 | 0.486 | 0.822 | 0.027 |
| 303 | 21088 | 0.384 | 0.757 | 0.028 |
| 304 | 21090 | 0.373 | 0.748 | 0.028 |
| 305 | 21087 | 0.389 | 0.762 | 0.028 |
| 306 | 21093 | 0.393 | 0.764 | 0.028 |
| 307 | 4491 | 0.326 | 0.672 | 0.028 |
| 308 | 4446 | 0.187 | 0.386 | 0.028 |
| 309 | 4445 | 0.191 | 0.391 | 0.028 |
| 310 | 1011 | 0.2 | 0.402 | 0.028 |
| 311 | 1091 | 0.191 | 0.391 | 0.028 |
| 312 | 11588 | 0.457 | 0.81 | 0.028 |
| 313 | 11589 | 0.448 | 0.806 | 0.028 |
| 314 | 11587 | 0.466 | 0.815 | 0.028 |
| 315 | 11585 | 0.48 | 0.823 | 0.028 |
| 316 | 28020 | 0.292 | 0.559 | 0.028 |
| 317 | 28022 | 0.294 | 0.561 | 0.028 |
| 318 | 28021 | 0.293 | 0.56 | 0.028 |
| 319 | 4582 | 0.27 | 0.539 | 0.028 |
| 320 | 4583 | 0.264 | 0.533 | 0.028 |
| 321 | 4581 | 0.276 | 0.545 | 0.028 |
| 322 | 1090 | 0.276 | 0.545 | 0.028 |
| 323 | 1010 | 0.288 | 0.561 | 0.028 |
| 324 | 4542 | 0.407 | 0.705 | 0.029 |
| 325 | 4541 | 0.416 | 0.712 | 0.029 |
| 326 | 11586 | 0.475 | 0.82 | 0.029 |
| 327 | 25555 | 0.352 | 0.752 | 0.029 |
| 328 | 20869 | 0.198 | 0.332 | 0.029 |
| 329 | 20925 | 0.146 | 0.343 | 0.029 |
| 330 | 20926 | 0.132 | 0.309 | 0.029 |
| 331 | 21170 | 0.101 | 0.248 | 0.029 |
| 332 | 21174 | 0.085 | 0.203 | 0.029 |
| 333 | 20870 | 0.193 | 0.329 | 0.029 |
| 334 | 14316 | 0.257 | 0.567 | 0.029 |
| 335 | 14312 | 0.255 | 0.564 | 0.029 |
| 336 | 14308 | 0.243 | 0.556 | 0.029 |
| 337 | 14302 | 0.235 | 0.548 | 0.029 |
| 338 | 14300 | 0.239 | 0.552 | 0.029 |
| 339 | 14310 | 0.256 | 0.565 | 0.029 |
| 340 | 14318 | 0.257 | 0.566 | 0.029 |
| 341 | 14306 | 0.247 | 0.559 | 0.029 |
| 342 | 25556 | 0.341 | 0.742 | 0.029 |
| 343 | 20871 | 0.188 | 0.326 | 0.029 |
| 344 | 14682 | 0.344 | 0.755 | 0.029 |
| 345 | 14680 | 0.35 | 0.76 | 0.029 |
| 346 | 14698 | 0.378 | 0.78 | 0.029 |
| 347 | 14696 | 0.379 | 0.781 | 0.029 |
| 348 | 14692 | 0.376 | 0.776 | 0.029 |
| 349 | 14690 | 0.377 | 0.778 | 0.029 |
| 350 | 14688 | 0.356 | 0.765 | 0.029 |
| 351 | 14686 | 0.362 | 0.77 | 0.029 |
| 352 | 21157 | 0.197 | 0.466 | 0.029 |
| 353 | 21161 | 0.19 | 0.457 | 0.029 |
| 354 | 21165 | 0.145 | 0.379 | 0.029 |
| 355 | 1302 | 0.567 | 0.88 | 0.029 |
| 356 | 21168 | 0.129 | 0.314 | 0.029 |
| 357 | 21172 | 0.116 | 0.277 | 0.029 |
| 358 | 4135 | 0.304 | 0.654 | 0.029 |
| 359 | 21160 | 0.192 | 0.46 | 0.029 |
| 360 | 21164 | 0.152 | 0.392 | 0.029 |
| 361 | 21156 | 0.197 | 0.467 | 0.029 |
| 362 | 21158 | 0.197 | 0.466 | 0.029 |
| 363 | 21162 | 0.189 | 0.455 | 0.029 |
| 364 | 21166 | 0.132 | 0.353 | 0.029 |
| 365 | 21173 | 0.104 | 0.249 | 0.029 |
| 366 | 21169 | 0.118 | 0.29 | 0.029 |
| 367 | 20759 | 0.546 | 0.871 | 0.029 |
| 368 | 14576 | 0.1 | 0.248 | 0.03 |
| 369 | 14556 | 0.177 | 0.435 | 0.03 |
| 370 | 14558 | 0.175 | 0.432 | 0.03 |
| 371 | 14560 | 0.183 | 0.44 | 0.03 |
| 372 | 14562 | 0.182 | 0.439 | 0.03 |
| 373 | 14564 | 0.184 | 0.442 | 0.03 |
| 374 | 14566 | 0.184 | 0.441 | 0.03 |
| 375 | 14552 | 0.17 | 0.427 | 0.03 |
| 376 | 14570 | 0.067 | 0.169 | 0.03 |
| 377 | 14550 | 0.173 | 0.43 | 0.03 |
| 378 | 14578 | 0.082 | 0.204 | 0.03 |
| 379 | 14580 | 0.057 | 0.131 | 0.03 |
| 380 | 14586 | 0.091 | 0.214 | 0.03 |
| 381 | 14588 | 0.072 | 0.168 | 0.03 |
| 382 | 14590 | 0.109 | 0.315 | 0.03 |
| 383 | 14596 | 0.132 | 0.361 | 0.03 |
| 384 | 14598 | 0.119 | 0.337 | 0.03 |
| 385 | 14568 | 0.183 | 0.44 | 0.03 |
| 386 | 20758 | 0.551 | 0.874 | 0.03 |
| 387 | 14350 | 0.305 | 0.668 | 0.03 |
| 388 | 14360 | 0.329 | 0.685 | 0.03 |
| 389 | 14362 | 0.328 | 0.683 | 0.03 |
| 390 | 14364 | 0.332 | 0.689 | 0.03 |
| 391 | 14366 | 0.331 | 0.687 | 0.03 |
| 392 | 14367 | 0.327 | 0.681 | 0.03 |
| 393 | 14356 | 0.316 | 0.677 | 0.03 |
| 394 | 14352 | 0.3 | 0.664 | 0.03 |
| 395 | 14358 | 0.31 | 0.673 | 0.03 |
| 396 | 14368 | 0.33 | 0.686 | 0.03 |
| 397 | 20760 | 0.541 | 0.869 | 0.03 |
| 398 | 4554 | 0.428 | 0.763 | 0.03 |
| 399 | 4553 | 0.439 | 0.771 | 0.03 |
| 400 | 19767 | 0.225 | 0.44 | 0.03 |
| 401 | 19777 | 0.225 | 0.44 | 0.03 |
| 402 | 14326 | 0.429 | 0.771 | 0.03 |
| 403 | 14324 | 0.44 | 0.777 | 0.03 |
| 404 | 14328 | 0.418 | 0.766 | 0.03 |
| 405 | 14408 | 0.343 | 0.765 | 0.031 |
| 406 | 14418 | 0.363 | 0.78 | 0.031 |
| 407 | 14417 | 0.359 | 0.774 | 0.031 |
| 408 | 14416 | 0.364 | 0.782 | 0.031 |
| 409 | 14414 | 0.365 | 0.783 | 0.031 |
| 410 | 14410 | 0.362 | 0.778 | 0.031 |
| 411 | 14406 | 0.348 | 0.77 | 0.031 |
| 412 | 14402 | 0.332 | 0.755 | 0.031 |
| 413 | 14400 | 0.337 | 0.76 | 0.031 |
| 414 | 14412 | 0.361 | 0.777 | 0.031 |
| 415 | 14440 | 0.157 | 0.317 | 0.031 |
| 416 | 14420 | 0.381 | 0.804 | 0.031 |
| 417 | 14423 | 0.406 | 0.823 | 0.031 |
| 418 | 14815 | 0.412 | 0.852 | 0.031 |
| 419 | 14425 | 0.398 | 0.817 | 0.031 |
| 420 | 14426 | 0.395 | 0.814 | 0.031 |
| 421 | 14428 | 0.388 | 0.809 | 0.031 |
| 422 | 14430 | 0.411 | 0.823 | 0.031 |
| 423 | 14432 | 0.41 | 0.822 | 0.031 |
| 424 | 14434 | 0.415 | 0.828 | 0.031 |
| 425 | 14813 | 0.415 | 0.855 | 0.031 |
| 426 | 14438 | 0.413 | 0.825 | 0.031 |
| 427 | 14422 | 0.374 | 0.798 | 0.031 |
| 428 | 14446 | 0.231 | 0.466 | 0.031 |
| 429 | 14448 | 0.191 | 0.384 | 0.031 |
| 430 | 14450 | 0.132 | 0.246 | 0.031 |
| 431 | 14456 | 0.208 | 0.4 | 0.031 |
| 432 | 14458 | 0.166 | 0.314 | 0.031 |
| 433 | 14460 | 0.219 | 0.586 | 0.031 |
| 434 | 14466 | 0.275 | 0.673 | 0.031 |
| 435 | 14818 | 0.408 | 0.846 | 0.031 |
| 436 | 14816 | 0.411 | 0.85 | 0.031 |
| 437 | 14436 | 0.414 | 0.827 | 0.031 |
| 438 | 14800 | 0.349 | 0.803 | 0.031 |
| 439 | 14810 | 0.403 | 0.841 | 0.031 |
| 440 | 14808 | 0.363 | 0.815 | 0.031 |
| 441 | 14806 | 0.376 | 0.826 | 0.031 |
| 442 | 14805 | 0.382 | 0.832 | 0.031 |
| 443 | 14804 | 0.389 | 0.837 | 0.031 |
| 444 | 14803 | 0.396 | 0.843 | 0.031 |
| 445 | 14424 | 0.402 | 0.82 | 0.031 |
| 446 | 14802 | 0.339 | 0.792 | 0.031 |
| 447 | 14412 | 0.361 | 0.777 | 0.031 |
| 448 | 14440 | 0.157 | 0.317 | 0.031 |
| 449 | 14420 | 0.381 | 0.804 | 0.031 |
| 450 | 14423 | 0.406 | 0.823 | 0.031 |
| 451 | 14815 | 0.412 | 0.852 | 0.031 |
| 452 | 14425 | 0.398 | 0.817 | 0.031 |
| 453 | 14426 | 0.395 | 0.814 | 0.031 |
| 454 | 14428 | 0.388 | 0.809 | 0.031 |
| 455 | 14430 | 0.411 | 0.823 | 0.031 |
| 456 | 14432 | 0.41 | 0.822 | 0.031 |
| 457 | 14434 | 0.415 | 0.828 | 0.031 |
| 458 | 14813 | 0.415 | 0.855 | 0.031 |
| 459 | 14438 | 0.413 | 0.825 | 0.031 |
| 460 | 14422 | 0.374 | 0.798 | 0.031 |
| 461 | 14446 | 0.231 | 0.466 | 0.031 |
| 462 | 14448 | 0.191 | 0.384 | 0.031 |
| 463 | 14450 | 0.132 | 0.246 | 0.031 |
| 464 | 14456 | 0.208 | 0.4 | 0.031 |
| 465 | 14458 | 0.166 | 0.314 | 0.031 |
| 466 | 14460 | 0.219 | 0.586 | 0.031 |
| 467 | 14466 | 0.275 | 0.673 | 0.031 |
| 468 | 14818 | 0.408 | 0.846 | 0.031 |
| 469 | 14816 | 0.411 | 0.85 | 0.031 |
| 470 | 14436 | 0.414 | 0.827 | 0.031 |
| 471 | 14800 | 0.349 | 0.803 | 0.031 |
| 472 | 14810 | 0.403 | 0.841 | 0.031 |
| 473 | 14808 | 0.363 | 0.815 | 0.031 |
| 474 | 14806 | 0.376 | 0.826 | 0.031 |
| 475 | 14805 | 0.382 | 0.832 | 0.031 |
| 476 | 14804 | 0.389 | 0.837 | 0.031 |
| 477 | 14803 | 0.396 | 0.843 | 0.031 |
| 478 | 14424 | 0.402 | 0.82 | 0.031 |
| 479 | 14802 | 0.339 | 0.792 | 0.031 |
| 480 | 14812 | 0.401 | 0.838 | 0.031 |
| 481 | 14468 | 0.244 | 0.628 | 0.031 |
| 482 | 14814 | 0.413 | 0.853 | 0.031 |
| 483 | 20747 | 0.18 | 0.378 | 0.031 |
| 484 | 20748 | 0.141 | 0.292 | 0.031 |
| 485 | 20749 | 0.111 | 0.225 | 0.031 |
| 486 | 4477 | 0.358 | 0.793 | 0.031 |
| 487 | 4478 | 0.353 | 0.787 | 0.031 |
| 488 | 4479 | 0.348 | 0.781 | 0.031 |
| 489 | 4482 | 0.348 | 0.781 | 0.031 |
| 490 | 4481 | 0.353 | 0.787 | 0.031 |
| 491 | 4480 | 0.358 | 0.793 | 0.031 |
| 492 | 4153 | 0.365 | 0.771 | 0.031 |
| 493 | 14338 | 0.26 | 0.559 | 0.031 |
| 494 | 14346 | 0.249 | 0.552 | 0.031 |
| 495 | 14342 | 0.238 | 0.541 | 0.031 |
| 496 | 14340 | 0.241 | 0.545 | 0.031 |
| 497 | 14337 | 0.257 | 0.555 | 0.031 |
| 498 | 14334 | 0.261 | 0.561 | 0.031 |
| 499 | 14330 | 0.259 | 0.558 | 0.031 |
| 500 | 14348 | 0.245 | 0.549 | 0.031 |
| 501 | 14336 | 0.26 | 0.56 | 0.031 |
| 502 | 14332 | 0.258 | 0.557 | 0.031 |
| 503 | 14398 | 0.255 | 0.567 | 0.031 |
| 504 | 14397 | 0.252 | 0.563 | 0.031 |
| 505 | 14396 | 0.255 | 0.568 | 0.031 |
| 506 | 14394 | 0.256 | 0.569 | 0.031 |
| 507 | 14392 | 0.253 | 0.565 | 0.031 |
| 508 | 14390 | 0.254 | 0.566 | 0.031 |
| 509 | 14382 | 0.234 | 0.549 | 0.031 |
| 510 | 14386 | 0.245 | 0.56 | 0.031 |
| 511 | 14388 | 0.241 | 0.557 | 0.031 |
| 512 | 14380 | 0.238 | 0.553 | 0.031 |
| 513 | 14665 | 0.353 | 0.737 | 0.031 |
| 514 | 14678 | 0.356 | 0.743 | 0.031 |
| 515 | 14676 | 0.357 | 0.744 | 0.031 |
| 516 | 14674 | 0.359 | 0.746 | 0.031 |
| 517 | 14670 | 0.355 | 0.741 | 0.031 |
| 518 | 14666 | 0.342 | 0.734 | 0.031 |
| 519 | 14672 | 0.354 | 0.74 | 0.031 |
| 520 | 14662 | 0.325 | 0.719 | 0.031 |
| 521 | 14660 | 0.33 | 0.724 | 0.031 |
| 522 | 14668 | 0.336 | 0.729 | 0.031 |
| 523 | 14664 | 0.35 | 0.744 | 0.031 |
| 524 | 14679 | 0.353 | 0.749 | 0.031 |
| 525 | 14675 | 0.355 | 0.752 | 0.031 |
| 526 | 14673 | 0.351 | 0.746 | 0.031 |
| 527 | 14669 | 0.333 | 0.735 | 0.031 |
| 528 | 14677 | 0.354 | 0.75 | 0.031 |
| 529 | 14667 | 0.339 | 0.74 | 0.031 |
| 530 | 14661 | 0.328 | 0.73 | 0.031 |
| 531 | 14663 | 0.322 | 0.725 | 0.031 |
| 532 | 14671 | 0.352 | 0.747 | 0.031 |
| 533 | 16661 | 0.429 | 0.803 | 0.032 |
| 534 | 4149 | 0.488 | 0.812 | 0.032 |
| 535 | 1322 | 0.59 | 0.871 | 0.032 |
| 536 | 14972 | 0.163 | 0.338 | 0.032 |
| 537 | 14970 | 0.163 | 0.338 | 0.032 |
| 538 | 14968 | 0.154 | 0.333 | 0.032 |
| 539 | 14966 | 0.157 | 0.335 | 0.032 |
| 540 | 14976 | 0.164 | 0.34 | 0.032 |
| 541 | 14962 | 0.149 | 0.329 | 0.032 |
| 542 | 14960 | 0.151 | 0.331 | 0.032 |
| 543 | 14978 | 0.164 | 0.339 | 0.032 |
| 544 | 4450 | 0.58 | 0.886 | 0.032 |
| 545 | 4449 | 0.593 | 0.894 | 0.032 |
| 546 | 4546 | 0.391 | 0.809 | 0.033 |
| 547 | 4544 | 0.391 | 0.809 | 0.033 |
| 548 | 4543 | 0.391 | 0.809 | 0.033 |
| 549 | 4545 | 0.391 | 0.809 | 0.033 |
| 550 | 4537 | 0.388 | 0.805 | 0.033 |
| 551 | 1323 | 0.59 | 0.872 | 0.033 |
| 552 | 4404 | 0.561 | 0.869 | 0.033 |
| 553 | 19768 | 0.307 | 0.567 | 0.033 |
| 554 | 4403 | 0.542 | 0.858 | 0.033 |
| 555 | 16664 | 0.364 | 0.747 | 0.033 |
| 556 | 4154 | 0.415 | 0.819 | 0.033 |
| 557 | 1313 | 0.535 | 0.846 | 0.033 |
| 558 | 6120 | 0.378 | 0.786 | 0.034 |
| 559 | 1314 | 0.528 | 0.846 | 0.034 |
| 560 | 20957 | 0.309 | 0.678 | 0.034 |
| 561 | 1303 | 0.567 | 0.884 | 0.034 |
| 562 | 14062 | 0.165 | 0.307 | 0.034 |
| 563 | 14786 | 0.37 | 0.808 | 0.034 |
| 564 | 14048 | 0.155 | 0.311 | 0.034 |
| 565 | 14058 | 0.379 | 0.786 | 0.034 |
| 566 | 14059 | 0.214 | 0.573 | 0.034 |
| 567 | 14798 | 0.405 | 0.828 | 0.034 |
| 568 | 14061 | 0.207 | 0.391 | 0.034 |
| 569 | 14045 | 0.23 | 0.455 | 0.034 |
| 570 | 14065 | 0.239 | 0.614 | 0.034 |
| 571 | 14067 | 0.131 | 0.241 | 0.034 |
| 572 | 14068 | 0.411 | 0.805 | 0.034 |
| 573 | 14069 | 0.414 | 0.808 | 0.034 |
| 574 | 14070 | 0.393 | 0.796 | 0.034 |
| 575 | 14060 | 0.269 | 0.658 | 0.034 |
| 576 | 14046 | 0.189 | 0.376 | 0.034 |
| 577 | 14169 | 0.372 | 0.781 | 0.034 |
| 578 | 14796 | 0.407 | 0.831 | 0.034 |
| 579 | 14788 | 0.357 | 0.797 | 0.034 |
| 580 | 14792 | 0.397 | 0.819 | 0.034 |
| 581 | 14782 | 0.332 | 0.775 | 0.034 |
| 582 | 14780 | 0.343 | 0.785 | 0.034 |
| 583 | 14793 | 0.411 | 0.836 | 0.034 |
| 584 | 14795 | 0.409 | 0.833 | 0.034 |
| 585 | 14794 | 0.41 | 0.835 | 0.034 |
| 586 | 14146 | 0.401 | 0.802 | 0.034 |
| 587 | 14166 | 0.41 | 0.803 | 0.034 |
| 588 | 14167 | 0.415 | 0.81 | 0.034 |
| 589 | 14071 | 0.386 | 0.791 | 0.034 |
| 590 | 14785 | 0.377 | 0.814 | 0.034 |
| 591 | 14790 | 0.399 | 0.823 | 0.034 |
| 592 | 14783 | 0.391 | 0.825 | 0.034 |
| 593 | 14784 | 0.384 | 0.819 | 0.034 |
| 594 | 14074 | 0.412 | 0.807 | 0.034 |
| 595 | 1312 | 0.555 | 0.856 | 0.035 |
| 596 | 1304 | 0.559 | 0.871 | 0.035 |
| 597 | 15014 | 0.379 | 0.798 | 0.035 |
| 598 | 25550 | 0.374 | 0.768 | 0.036 |
| 599 | 16681 | 0.608 | 0.889 | 0.036 |
| 600 | 25540 | 0.295 | 0.647 | 0.036 |
| 601 | 15012 | 0.385 | 0.778 | 0.036 |
| 602 | 15008 | 0.385 | 0.786 | 0.036 |
| 603 | 16682 | 0.603 | 0.881 | 0.037 |
| 604 | 1316 | 0.496 | 0.825 | 0.037 |
| 605 | 25552 | 0.252 | 0.552 | 0.037 |
| 606 | 25553 | 0.246 | 0.542 | 0.037 |
| 607 | 25561 | 0.384 | 0.749 | 0.037 |
| 608 | 1305 | 0.512 | 0.846 | 0.037 |
| 609 | 4549 | 0.39 | 0.797 | 0.038 |
| 610 | 4550 | 0.384 | 0.79 | 0.038 |
| 611 | 4547 | 0.403 | 0.811 | 0.038 |
| 612 | 4548 | 0.397 | 0.804 | 0.038 |
| 613 | 4574 | 0.193 | 0.445 | 0.038 |
| 614 | 4573 | 0.23 | 0.519 | 0.038 |
| 615 | 4538 | 0.4 | 0.808 | 0.038 |
| 616 | 25557 | 0.367 | 0.761 | 0.038 |
| 617 | 16680 | 0.614 | 0.885 | 0.038 |
| 618 | 19778 | 0.362 | 0.652 | 0.038 |
| 619 | 19769 | 0.362 | 0.652 | 0.038 |
| 620 | 15009 | 0.384 | 0.78 | 0.038 |
| 621 | 4556 | 0.632 | 0.905 | 0.038 |
| 622 | 4558 | 0.597 | 0.885 | 0.038 |
| 623 | 4557 | 0.61 | 0.893 | 0.038 |
| 624 | 1315 | 0.512 | 0.837 | 0.038 |
| 625 | 20888 | 0.211 | 0.446 | 0.038 |
| 626 | 11576 | 0.627 | 0.89 | 0.039 |
| 627 | 11577 | 0.622 | 0.887 | 0.039 |
| 628 | 20890 | 0.203 | 0.439 | 0.039 |
| 629 | 11563 | 0.591 | 0.884 | 0.039 |
| 630 | 11560 | 0.618 | 0.893 | 0.039 |
| 631 | 11565 | 0.564 | 0.875 | 0.039 |
| 632 | 11564 | 0.577 | 0.88 | 0.039 |
| 633 | 11562 | 0.604 | 0.889 | 0.039 |
| 634 | 11561 | 0.611 | 0.891 | 0.039 |
| 635 | 25543 | 0.252 | 0.476 | 0.039 |
| 636 | 3095 | 0.337 | 0.73 | 0.039 |
| 637 | 3093 | 0.344 | 0.737 | 0.039 |
| 638 | 3094 | 0.34 | 0.734 | 0.039 |
| 639 | 11573 | 0.638 | 0.897 | 0.039 |
| 640 | 20889 | 0.207 | 0.443 | 0.039 |
| 641 | 21085 | 0.379 | 0.753 | 0.039 |
| 642 | 16685 | 0.581 | 0.873 | 0.039 |
| 643 | 11574 | 0.635 | 0.895 | 0.039 |
| 644 | 21084 | 0.384 | 0.758 | 0.039 |
| 645 | 21083 | 0.39 | 0.763 | 0.039 |
| 646 | 21086 | 0.373 | 0.748 | 0.039 |
| 647 | 21431 | 0.636 | 0.9 | 0.039 |
| 648 | 21422 | 0.636 | 0.902 | 0.039 |
| 649 | 21426 | 0.597 | 0.885 | 0.039 |
| 650 | 21429 | 0.647 | 0.906 | 0.04 |
| 651 | 21424 | 0.622 | 0.896 | 0.04 |
| 652 | 21421 | 0.643 | 0.905 | 0.04 |
| 653 | 21425 | 0.609 | 0.89 | 0.04 |
| 654 | 21428 | 0.653 | 0.908 | 0.04 |
| 655 | 21423 | 0.628 | 0.898 | 0.04 |
| 656 | 21430 | 0.642 | 0.903 | 0.04 |
| 657 | 11575 | 0.631 | 0.893 | 0.04 |
| 658 | 11572 | 0.64 | 0.898 | 0.04 |
| 659 | 4370 | 0.572 | 0.86 | 0.04 |
| 660 | 4374 | 0.552 | 0.851 | 0.04 |
| 661 | 4375 | 0.614 | 0.88 | 0.04 |
| 662 | 4373 | 0.592 | 0.87 | 0.04 |
| 663 | 25548 | 0.402 | 0.695 | 0.04 |
| 664 | 1325 | 0.547 | 0.85 | 0.041 |
| 665 | 11581 | 0.648 | 0.891 | 0.041 |
| 666 | 11580 | 0.652 | 0.894 | 0.041 |
| 667 | 11570 | 0.591 | 0.878 | 0.041 |
| 668 | 11569 | 0.605 | 0.883 | 0.041 |
| 669 | 11566 | 0.634 | 0.892 | 0.041 |
| 670 | 11571 | 0.578 | 0.874 | 0.041 |
| 671 | 11568 | 0.619 | 0.887 | 0.041 |
| 672 | 11567 | 0.627 | 0.889 | 0.041 |
| 673 | 11583 | 0.639 | 0.885 | 0.041 |
| 674 | 11582 | 0.643 | 0.888 | 0.042 |
| 675 | 11578 | 0.657 | 0.897 | 0.042 |
| 676 | 11579 | 0.655 | 0.895 | 0.042 |
| 677 | 25549 | 0.384 | 0.757 | 0.042 |
| 678 | 4454 | 0.624 | 0.901 | 0.042 |
| 679 | 4453 | 0.639 | 0.909 | 0.042 |
| 680 | 1324 | 0.575 | 0.867 | 0.043 |
| 681 | 4452 | 0.601 | 0.892 | 0.043 |
| 682 | 4451 | 0.615 | 0.9 | 0.043 |
| 683 | 20908 | 0.15 | 0.334 | 0.045 |
| 684 | 3354 | 0.467 | 0.774 | 0.045 |
| 685 | 3350 | 0.494 | 0.787 | 0.045 |
| 686 | 3353 | 0.475 | 0.778 | 0.045 |
| 687 | 3352 | 0.481 | 0.781 | 0.045 |
| 688 | 3351 | 0.488 | 0.784 | 0.045 |
| 689 | 26171 | 0.458 | 0.776 | 0.045 |
| 690 | 26174 | 0.449 | 0.77 | 0.045 |
| 691 | 26172 | 0.463 | 0.779 | 0.045 |
| 692 | 26173 | 0.446 | 0.769 | 0.045 |
| 693 | 20755 | 0.673 | 0.911 | 0.046 |
| 694 | 14520 | 0.075 | 0.135 | 0.046 |
| 695 | 14548 | 0.103 | 0.267 | 0.046 |
| 696 | 14546 | 0.119 | 0.286 | 0.046 |
| 697 | 14540 | 0.091 | 0.249 | 0.046 |
| 698 | 14538 | 0.079 | 0.133 | 0.046 |
| 699 | 14536 | 0.1 | 0.169 | 0.046 |
| 700 | 14530 | 0.063 | 0.104 | 0.046 |
| 701 | 14528 | 0.091 | 0.163 | 0.046 |
| 702 | 14526 | 0.111 | 0.197 | 0.046 |
| 703 | 14508 | 0.185 | 0.344 | 0.046 |
| 704 | 14506 | 0.19 | 0.347 | 0.046 |
| 705 | 14502 | 0.176 | 0.34 | 0.046 |
| 706 | 14500 | 0.181 | 0.342 | 0.046 |
| 707 | 20928 | 0.114 | 0.252 | 0.046 |
| 708 | 20754 | 0.681 | 0.914 | 0.046 |
| 709 | 20756 | 0.666 | 0.908 | 0.046 |
| 710 | 20848 | 0.539 | 0.855 | 0.046 |
| 711 | 20852 | 0.582 | 0.874 | 0.046 |
| 712 | 20851 | 0.591 | 0.878 | 0.046 |
| 713 | 20842 | 0.581 | 0.878 | 0.047 |
| 714 | 9797 | 0.581 | 0.878 | 0.047 |
| 715 | 20849 | 0.573 | 0.87 | 0.047 |
| 716 | 20840 | 0.596 | 0.885 | 0.047 |
| 717 | 9793 | 0.596 | 0.885 | 0.047 |
| 718 | 9795 | 0.588 | 0.882 | 0.047 |
| 719 | 20841 | 0.588 | 0.882 | 0.047 |
| 720 | 21484 | 0.587 | 0.883 | 0.047 |
| 721 | 20850 | 0.6 | 0.882 | 0.047 |
| 722 | 20847 | 0.556 | 0.863 | 0.047 |
| 723 | 21486 | 0.575 | 0.877 | 0.047 |
| 724 | 25551 | 0.25 | 0.477 | 0.047 |
| 725 | 21483 | 0.593 | 0.886 | 0.047 |
| 726 | 21485 | 0.58 | 0.88 | 0.047 |
| 727 | 21487 | 0.564 | 0.872 | 0.047 |
| 728 | 20909 | 0.146 | 0.332 | 0.047 |
| 729 | 20738 | 0.171 | 0.349 | 0.047 |
| 730 | 20844 | 0.597 | 0.882 | 0.047 |
| 731 | 9794 | 0.597 | 0.882 | 0.047 |
| 732 | 20735 | 0.174 | 0.353 | 0.047 |
| 733 | 20845 | 0.589 | 0.878 | 0.047 |
| 734 | 9796 | 0.589 | 0.878 | 0.047 |
| 735 | 20751 | 0.693 | 0.918 | 0.047 |
| 736 | 20750 | 0.7 | 0.92 | 0.048 |
| 737 | 20752 | 0.677 | 0.912 | 0.048 |
| 738 | 20846 | 0.581 | 0.874 | 0.048 |
| 739 | 9798 | 0.581 | 0.874 | 0.048 |
| 740 | 25545 | 0.327 | 0.567 | 0.048 |
| 741 | 11375 | 0.596 | 0.861 | 0.048 |
| 742 | 11373 | 0.62 | 0.87 | 0.048 |
| 743 | 11374 | 0.649 | 0.88 | 0.048 |
| 744 | 25554 | 0.407 | 0.685 | 0.048 |
| 745 | 20929 | 0.103 | 0.227 | 0.048 |
| 746 | 25547 | 0.311 | 0.551 | 0.049 |
| 747 | 25544 | 0.354 | 0.59 | 0.05 |
| 748 | 15015 | 0.624 | 0.885 | 0.05 |
| 749 | 25546 | 0.32 | 0.559 | 0.05 |
| 750 | 15011 | 0.57 | 0.875 | 0.05 |
| 751 | 21342 | 0.155 | 0.358 | 0.05 |
| 752 | 21326 | 0.134 | 0.306 | 0.05 |
| 753 | 21495 | 0.604 | 0.893 | 0.05 |
| 754 | 21434 | 0.642 | 0.901 | 0.05 |
| 755 | 21440 | 0.659 | 0.908 | 0.05 |
| 756 | 21436 | 0.629 | 0.896 | 0.05 |
| 757 | 21442 | 0.648 | 0.903 | 0.05 |
| 758 | 21337 | 0.268 | 0.465 | 0.05 |
| 759 | 21349 | 0.152 | 0.253 | 0.05 |
| 760 | 21345 | 0.148 | 0.295 | 0.05 |
| 761 | 21333 | 0.137 | 0.217 | 0.05 |
| 762 | 21321 | 0.237 | 0.399 | 0.05 |
| 763 | 21329 | 0.128 | 0.252 | 0.05 |
| 764 | 21340 | 0.19 | 0.398 | 0.05 |
| 765 | 21438 | 0.603 | 0.885 | 0.05 |
| 766 | 21496 | 0.592 | 0.887 | 0.05 |
| 767 | 21433 | 0.649 | 0.905 | 0.05 |
| 768 | 21324 | 0.165 | 0.341 | 0.05 |
| 769 | 21435 | 0.635 | 0.898 | 0.05 |
| 770 | 21441 | 0.654 | 0.905 | 0.05 |
| 771 | 21443 | 0.642 | 0.9 | 0.05 |
| 772 | 21437 | 0.615 | 0.89 | 0.05 |
| 773 | 21336 | 0.273 | 0.468 | 0.05 |
| 774 | 21348 | 0.169 | 0.28 | 0.05 |
| 775 | 21344 | 0.163 | 0.319 | 0.05 |
| 776 | 21328 | 0.143 | 0.273 | 0.05 |
| 777 | 21332 | 0.151 | 0.24 | 0.05 |
| 778 | 21320 | 0.242 | 0.402 | 0.05 |
| 779 | 21338 | 0.264 | 0.463 | 0.05 |
| 780 | 21346 | 0.122 | 0.252 | 0.05 |
| 781 | 21350 | 0.125 | 0.205 | 0.05 |
| 782 | 21322 | 0.234 | 0.397 | 0.05 |
| 783 | 21330 | 0.105 | 0.215 | 0.05 |
| 784 | 21334 | 0.111 | 0.176 | 0.05 |
| 785 | 21341 | 0.177 | 0.385 | 0.051 |
| 786 | 16660 | 0.489 | 0.819 | 0.051 |
| 787 | 21325 | 0.155 | 0.329 | 0.051 |
| 788 | 4132 | 0.539 | 0.856 | 0.051 |
| 789 | 1296 | 0.574 | 0.878 | 0.051 |
| 790 | 20910 | 0.143 | 0.33 | 0.051 |
| 791 | 19772 | 0.115 | 0.186 | 0.051 |
| 792 | 14250 | 0.178 | 0.322 | 0.051 |
| 793 | 14252 | 0.173 | 0.32 | 0.051 |
| 794 | 14376 | 0.2 | 0.331 | 0.051 |
| 795 | 14370 | 0.198 | 0.33 | 0.051 |
| 796 | 14268 | 0.088 | 0.153 | 0.051 |
| 797 | 14256 | 0.187 | 0.327 | 0.051 |
| 798 | 14258 | 0.182 | 0.325 | 0.051 |
| 799 | 14372 | 0.198 | 0.329 | 0.051 |
| 800 | 14276 | 0.098 | 0.16 | 0.051 |
| 801 | 14260 | 0.072 | 0.126 | 0.051 |
| 802 | 14286 | 0.116 | 0.27 | 0.051 |
| 803 | 14278 | 0.078 | 0.125 | 0.051 |
| 804 | 14288 | 0.101 | 0.252 | 0.051 |
| 805 | 14270 | 0.061 | 0.098 | 0.051 |
| 806 | 14266 | 0.108 | 0.185 | 0.051 |
| 807 | 14378 | 0.199 | 0.331 | 0.051 |
| 808 | 14280 | 0.09 | 0.236 | 0.051 |
| 809 | 4580 | 0.282 | 0.543 | 0.052 |
| 810 | 4578 | 0.307 | 0.558 | 0.052 |
| 811 | 4579 | 0.294 | 0.55 | 0.052 |
| 812 | 13112 | 0.553 | 0.874 | 0.052 |
| 813 | 13110 | 0.581 | 0.887 | 0.052 |
| 814 | 13111 | 0.567 | 0.88 | 0.052 |
| 815 | 13101 | 0.612 | 0.895 | 0.052 |
| 816 | 19770 | 0.476 | 0.778 | 0.052 |
| 817 | 25542 | 0.515 | 0.798 | 0.052 |
| 818 | 28029 | 0.62 | 0.909 | 0.053 |
| 819 | 1293 | 0.63 | 0.899 | 0.053 |
| 820 | 28028 | 0.623 | 0.911 | 0.053 |
| 821 | 4555 | 0.608 | 0.896 | 0.053 |
| 822 | 4407 | 0.522 | 0.858 | 0.053 |
| 823 | 4406 | 0.548 | 0.87 | 0.053 |
| 824 | 14734 | 0.658 | 0.908 | 0.053 |
| 825 | 14737 | 0.642 | 0.898 | 0.053 |
| 826 | 14726 | 0.605 | 0.893 | 0.053 |
| 827 | 14725 | 0.614 | 0.896 | 0.053 |
| 828 | 14728 | 0.587 | 0.887 | 0.053 |
| 829 | 14724 | 0.623 | 0.899 | 0.053 |
| 830 | 14730 | 0.649 | 0.903 | 0.053 |
| 831 | 14732 | 0.646 | 0.901 | 0.053 |
| 832 | 14723 | 0.633 | 0.902 | 0.053 |
| 833 | 14722 | 0.555 | 0.875 | 0.053 |
| 834 | 14720 | 0.571 | 0.881 | 0.053 |
| 835 | 14733 | 0.66 | 0.909 | 0.053 |
| 836 | 14736 | 0.655 | 0.906 | 0.053 |
| 837 | 14735 | 0.657 | 0.907 | 0.053 |
| 838 | 14738 | 0.652 | 0.905 | 0.053 |
| 839 | 20737 | 0.169 | 0.326 | 0.053 |
| 840 | 20731 | 0.152 | 0.327 | 0.053 |
| 841 | 20732 | 0.173 | 0.325 | 0.053 |
| 842 | 21476 | 0.575 | 0.878 | 0.054 |
| 843 | 20736 | 0.15 | 0.326 | 0.054 |
| 844 | 21478 | 0.553 | 0.866 | 0.054 |
| 845 | 1294 | 0.617 | 0.893 | 0.054 |
| 846 | 4133 | 0.569 | 0.88 | 0.054 |
| 847 | 21477 | 0.563 | 0.872 | 0.054 |
| 848 | 21481 | 0.593 | 0.886 | 0.055 |
| 849 | 19773 | 0.185 | 0.288 | 0.055 |
| 850 | 14214 | 0.638 | 0.909 | 0.055 |
| 851 | 14843 | 0.575 | 0.893 | 0.055 |
| 852 | 14844 | 0.555 | 0.886 | 0.055 |
| 853 | 14748 | 0.504 | 0.895 | 0.055 |
| 854 | 14746 | 0.53 | 0.907 | 0.055 |
| 855 | 14213 | 0.639 | 0.909 | 0.055 |
| 856 | 14215 | 0.637 | 0.908 | 0.055 |
| 857 | 14216 | 0.635 | 0.907 | 0.055 |
| 858 | 14212 | 0.627 | 0.901 | 0.055 |
| 859 | 14154 | 0.542 | 0.876 | 0.055 |
| 860 | 14758 | 0.607 | 0.929 | 0.055 |
| 861 | 14151 | 0.588 | 0.893 | 0.055 |
| 862 | 14210 | 0.63 | 0.903 | 0.055 |
| 863 | 14753 | 0.621 | 0.939 | 0.055 |
| 864 | 14755 | 0.615 | 0.935 | 0.055 |
| 865 | 14148 | 0.614 | 0.903 | 0.055 |
| 866 | 14218 | 0.632 | 0.905 | 0.055 |
| 867 | 14149 | 0.605 | 0.9 | 0.055 |
| 868 | 14752 | 0.592 | 0.92 | 0.055 |
| 869 | 14150 | 0.597 | 0.896 | 0.055 |
| 870 | 14153 | 0.556 | 0.882 | 0.055 |
| 871 | 14152 | 0.572 | 0.887 | 0.055 |
| 872 | 14750 | 0.597 | 0.924 | 0.055 |
| 873 | 14240 | 0.263 | 0.643 | 0.055 |
| 874 | 14743 | 0.575 | 0.926 | 0.055 |
| 875 | 14742 | 0.454 | 0.87 | 0.055 |
| 876 | 14744 | 0.559 | 0.92 | 0.055 |
| 877 | 14248 | 0.301 | 0.689 | 0.055 |
| 878 | 14220 | 0.234 | 0.349 | 0.055 |
| 879 | 14740 | 0.476 | 0.882 | 0.055 |
| 880 | 14756 | 0.613 | 0.933 | 0.055 |
| 881 | 14238 | 0.25 | 0.345 | 0.055 |
| 882 | 14745 | 0.544 | 0.913 | 0.055 |
| 883 | 14236 | 0.316 | 0.439 | 0.055 |
| 884 | 14754 | 0.618 | 0.937 | 0.055 |
| 885 | 14230 | 0.199 | 0.27 | 0.055 |
| 886 | 14228 | 0.285 | 0.422 | 0.055 |
| 887 | 14226 | 0.349 | 0.511 | 0.055 |
| 888 | 14246 | 0.35 | 0.738 | 0.055 |
| 889 | 6700 | 0.361 | 0.539 | 0.055 |
| 890 | 4134 | 0.527 | 0.86 | 0.055 |
| 891 | 25541 | 0.525 | 0.804 | 0.055 |
| 892 | 5225 | 0.483 | 0.817 | 0.055 |
| 893 | 5224 | 0.493 | 0.822 | 0.055 |
| 894 | 5223 | 0.501 | 0.826 | 0.055 |
| 895 | 5222 | 0.508 | 0.829 | 0.055 |
| 896 | 5221 | 0.52 | 0.834 | 0.055 |
| 897 | 15013 | 0.577 | 0.868 | 0.055 |
| 898 | 21482 | 0.581 | 0.88 | 0.056 |
| 899 | 21475 | 0.587 | 0.883 | 0.056 |
| 900 | 1295 | 0.605 | 0.888 | 0.056 |
| 901 | 15010 | 0.585 | 0.875 | 0.057 |
| 902 | 4575 | 0.182 | 0.327 | 0.058 |
| 903 | 4576 | 0.174 | 0.322 | 0.058 |
| 904 | 20800 | 0.26 | 0.378 | 0.058 |
| 905 | 4577 | 0.167 | 0.318 | 0.058 |
| 906 | 20801 | 0.255 | 0.376 | 0.059 |
| 907 | 11479 | 0.327 | 0.517 | 0.06 |
| 908 | 11477 | 0.317 | 0.513 | 0.06 |
| 909 | 11478 | 0.322 | 0.515 | 0.06 |
| 910 | 11473 | 0.297 | 0.504 | 0.06 |
| 911 | 21419 | 0.688 | 0.884 | 0.061 |
| 912 | 21418 | 0.705 | 0.89 | 0.061 |
| 913 | 1297 | 0.575 | 0.877 | 0.061 |
| 914 | 20953 | 0.181 | 0.453 | 0.063 |
| 915 | 20950 | 0.185 | 0.236 | 0.063 |
| 916 | 20954 | 0.179 | 0.449 | 0.064 |
| 917 | 4563 | 0.719 | 0.908 | 0.066 |
| 918 | 4564 | 0.715 | 0.907 | 0.066 |
| 919 | 4565 | 0.711 | 0.905 | 0.066 |
| 920 | 4552 | 0.694 | 0.899 | 0.066 |
| 921 | 4551 | 0.702 | 0.902 | 0.066 |
| 922 | 20952 | 0.184 | 0.457 | 0.066 |
| 923 | 19774 | 0.247 | 0.394 | 0.069 |
| 924 | 16856 | 0.598 | 0.856 | 0.07 |
| 925 | 11166 | 0.321 | 0.504 | 0.072 |
| 926 | 11165 | 0.331 | 0.508 | 0.072 |
| 927 | 11167 | 0.309 | 0.499 | 0.072 |
| 928 | 16855 | 0.615 | 0.862 | 0.073 |
| 929 | 21453 | 0.664 | 0.856 | 0.081 |
| 930 | 21451 | 0.696 | 0.868 | 0.081 |
| 931 | 21450 | 0.714 | 0.874 | 0.082 |
| 932 | 3259 | 0.701 | 0.88 | 0.082 |
| 933 | 3257 | 0.723 | 0.886 | 0.082 |
| 934 | 3258 | 0.713 | 0.883 | 0.082 |
| 935 | 3260 | 0.69 | 0.877 | 0.082 |
| 936 | 3261 | 0.677 | 0.874 | 0.082 |
| 937 | 21452 | 0.68 | 0.862 | 0.082 |
| 938 | 20960 | 0.168 | 0.262 | 0.085 |
| 939 | 11487 | 0.308 | 0.508 | 0.086 |
| 940 | 21457 | 0.715 | 0.887 | 0.086 |
| 941 | 21456 | 0.733 | 0.893 | 0.086 |
| 942 | 21353 | 0.187 | 0.277 | 0.087 |
| 943 | 21352 | 0.191 | 0.278 | 0.087 |
| 944 | 21354 | 0.183 | 0.275 | 0.087 |
| 945 | 21455 | 0.742 | 0.896 | 0.087 |
| 946 | 20864 | 0.574 | 0.844 | 0.087 |
| 947 | 20867 | 0.532 | 0.825 | 0.087 |
| 948 | 20866 | 0.548 | 0.833 | 0.087 |
| 949 | 20865 | 0.565 | 0.84 | 0.087 |
| 950 | 20863 | 0.583 | 0.848 | 0.088 |
| 951 | 3402 | 0.613 | 0.855 | 0.089 |
| 952 | 26116 | 0.67 | 0.873 | 0.089 |
| 953 | 3401 | 0.632 | 0.861 | 0.089 |
| 954 | 3400 | 0.657 | 0.869 | 0.089 |
| 955 | 20836 | 0.298 | 0.533 | 0.092 |
| 956 | 20837 | 0.335 | 0.463 | 0.092 |
| 957 | 20835 | 0.357 | 0.696 | 0.093 |
| 958 | 3247 | 0.684 | 0.885 | 0.095 |
| 959 | 3239 | 0.695 | 0.889 | 0.095 |
| 960 | 3238 | 0.708 | 0.892 | 0.095 |
| 961 | 3237 | 0.719 | 0.895 | 0.095 |
| 962 | 3248 | 0.67 | 0.881 | 0.095 |
| 963 | 16871 | 0.539 | 0.784 | 0.096 |
| 964 | 4327 | 0.715 | 0.898 | 0.097 |
| 965 | 4328 | 0.7 | 0.892 | 0.097 |
| 966 | 16870 | 0.552 | 0.789 | 0.097 |
| 967 | 20122 | 0.412 | 0.629 | 0.099 |
| 968 | 16850 | 0.609 | 0.842 | 0.103 |
| 969 | 20124 | 0.398 | 0.642 | 0.103 |
| 970 | 20123 | 0.422 | 0.653 | 0.106 |
| 971 | 16851 | 0.608 | 0.841 | 0.107 |
| 972 | 20120 | 0.467 | 0.663 | 0.109 |
| 973 | 19797 | 0.506 | 0.801 | 0.11 |
| 974 | 19771 | 0.625 | 0.875 | 0.11 |
| 975 | 19776 | 0.506 | 0.801 | 0.11 |
| 976 | 19779 | 0.625 | 0.875 | 0.11 |
| 977 | 5234 | 0.652 | 0.861 | 0.113 |
| 978 | 5235 | 0.635 | 0.856 | 0.113 |
| 979 | 5232 | 0.678 | 0.868 | 0.113 |
| 980 | 5231 | 0.698 | 0.873 | 0.113 |
| 981 | 5233 | 0.666 | 0.865 | 0.113 |
| 982 | 5237 | 0.715 | 0.876 | 0.114 |
| 983 | 5238 | 0.71 | 0.875 | 0.114 |
| 984 | 5239 | 0.703 | 0.873 | 0.114 |
| 985 | 4348 | 0.662 | 0.798 | 0.114 |
| 986 | 4343 | 0.637 | 0.789 | 0.114 |
| 987 | 20121 | 0.44 | 0.638 | 0.114 |
| 988 | 5229 | 0.728 | 0.879 | 0.115 |
| 989 | 5230 | 0.724 | 0.878 | 0.115 |
| 990 | 5228 | 0.73 | 0.88 | 0.115 |
| 991 | 5227 | 0.733 | 0.88 | 0.115 |
| 992 | 21212 | 0.18 | 0.355 | 0.123 |
| 993 | 21213 | 0.168 | 0.343 | 0.124 |
| 994 | 21214 | 0.146 | 0.32 | 0.124 |
| 995 | 21222 | 0.124 | 0.184 | 0.124 |
| 996 | 20861 | 0.593 | 0.83 | 0.124 |
| 997 | 21216 | 0.155 | 0.285 | 0.124 |
| 998 | 21218 | 0.115 | 0.225 | 0.124 |
| 999 | 21389 | 0.272 | 0.402 | 0.124 |
| 1000 | 21209 | 0.261 | 0.415 | 0.124 |
| 1001 | 21220 | 0.167 | 0.25 | 0.124 |
| 1002 | 21208 | 0.266 | 0.417 | 0.124 |
| 1003 | 21217 | 0.139 | 0.263 | 0.124 |
| 1004 | 20862 | 0.58 | 0.825 | 0.124 |
| 1005 | 21388 | 0.278 | 0.405 | 0.124 |
| 1006 | 21210 | 0.256 | 0.413 | 0.124 |
| 1007 | 21390 | 0.266 | 0.399 | 0.124 |
| 1008 | 21221 | 0.151 | 0.225 | 0.124 |
| 1009 | 21202 | 0.152 | 0.286 | 0.131 |
| 1010 | 21197 | 0.223 | 0.434 | 0.131 |
| 1011 | 21193 | 0.355 | 0.525 | 0.131 |
| 1012 | 21205 | 0.207 | 0.286 | 0.131 |
| 1013 | 20932 | 0.247 | 0.355 | 0.131 |
| 1014 | 21200 | 0.209 | 0.361 | 0.131 |
| 1015 | 20931 | 0.276 | 0.394 | 0.131 |
| 1016 | 21192 | 0.364 | 0.528 | 0.131 |
| 1017 | 21204 | 0.231 | 0.318 | 0.131 |
| 1018 | 21196 | 0.241 | 0.45 | 0.131 |
| 1019 | 21198 | 0.192 | 0.405 | 0.131 |
| 1020 | 21206 | 0.169 | 0.233 | 0.131 |
| 1021 | 21194 | 0.348 | 0.521 | 0.131 |
| 1022 | 21201 | 0.187 | 0.334 | 0.131 |
| 1023 | 20935 | 0.315 | 0.429 | 0.132 |
| 1024 | 21184 | 0.261 | 0.436 | 0.132 |
| 1025 | 21185 | 0.234 | 0.403 | 0.132 |
| 1026 | 21186 | 0.188 | 0.344 | 0.132 |
| 1027 | 21182 | 0.237 | 0.489 | 0.132 |
| 1028 | 21177 | 0.455 | 0.635 | 0.132 |
| 1029 | 21189 | 0.267 | 0.347 | 0.132 |
| 1030 | 21180 | 0.302 | 0.544 | 0.132 |
| 1031 | 20934 | 0.352 | 0.476 | 0.132 |
| 1032 | 21188 | 0.296 | 0.386 | 0.132 |
| 1033 | 21176 | 0.465 | 0.639 | 0.132 |
| 1034 | 21178 | 0.444 | 0.631 | 0.132 |
| 1035 | 21190 | 0.219 | 0.283 | 0.132 |
| 1036 | 21181 | 0.278 | 0.525 | 0.132 |
| 1037 | 21317 | 0.213 | 0.346 | 0.136 |
| 1038 | 21316 | 0.217 | 0.348 | 0.136 |
| 1039 | 21318 | 0.209 | 0.344 | 0.136 |
| 1040 | 20118 | 0.408 | 0.699 | 0.141 |
| 1041 | 20116 | 0.474 | 0.743 | 0.141 |
| 1042 | 21446 | 0.576 | 0.77 | 0.145 |
| 1043 | 21448 | 0.563 | 0.764 | 0.145 |
| 1044 | 21445 | 0.582 | 0.772 | 0.145 |
| 1045 | 21447 | 0.57 | 0.767 | 0.145 |
| 1046 | 9929 | 0.741 | 0.826 | 0.146 |
| 1047 | 28602 | 0.507 | 0.744 | 0.146 |
| 1048 | 9928 | 0.747 | 0.838 | 0.146 |
| 1049 | 20117 | 0.392 | 0.654 | 0.147 |
| 1050 | 1035 | 0.67 | 0.827 | 0.148 |
| 1051 | 1031 | 0.727 | 0.845 | 0.148 |
| 1052 | 1032 | 0.688 | 0.816 | 0.148 |
| 1053 | 1033 | 0.689 | 0.825 | 0.148 |
| 1054 | 1034 | 0.675 | 0.825 | 0.148 |
| 1055 | 18101 | 0.672 | 0.864 | 0.149 |
| 1056 | 18102 | 0.654 | 0.86 | 0.149 |
| 1057 | 18103 | 0.644 | 0.855 | 0.149 |
| 1058 | 18104 | 0.626 | 0.85 | 0.149 |
| 1059 | 18100 | 0.684 | 0.868 | 0.149 |
| 1060 | 9741 | 0.655 | 0.814 | 0.152 |
| 1061 | 4146 | 0.692 | 0.827 | 0.157 |
| 1062 | 9925 | 0.622 | 0.807 | 0.157 |
| 1063 | 9926 | 0.589 | 0.796 | 0.157 |
| 1064 | 9930 | 0.75 | 0.83 | 0.157 |
| 1065 | 9924 | 0.662 | 0.819 | 0.157 |
| 1066 | 9923 | 0.676 | 0.826 | 0.158 |
| 1067 | 20101 | 0.692 | 0.823 | 0.159 |
| 1068 | 20111 | 0.497 | 0.761 | 0.159 |
| 1069 | 4147 | 0.674 | 0.816 | 0.159 |
| 1070 | 4145 | 0.713 | 0.835 | 0.159 |
| 1071 | 20119 | 0.356 | 0.631 | 0.16 |
| 1072 | 9739 | 0.685 | 0.827 | 0.16 |
| 1073 | 20112 | 0.423 | 0.724 | 0.161 |
| 1074 | 20102 | 0.706 | 0.829 | 0.162 |
| 1075 | 20115 | 0.413 | 0.669 | 0.162 |
| 1076 | 4144 | 0.728 | 0.838 | 0.162 |
| 1077 | 20100 | 0.712 | 0.835 | 0.163 |
| 1078 | 21304 | 0.033 | 0.061 | 0.163 |
| 1079 | 21305 | 0.029 | 0.056 | 0.163 |
| 1080 | 21306 | 0.023 | 0.047 | 0.163 |
| 1081 | 21302 | 0.03 | 0.068 | 0.163 |
| 1082 | 9740 | 0.666 | 0.815 | 0.163 |
| 1083 | 21309 | 0.031 | 0.048 | 0.163 |
| 1084 | 21297 | 0.054 | 0.089 | 0.163 |
| 1085 | 20103 | 0.674 | 0.817 | 0.163 |
| 1086 | 21300 | 0.038 | 0.076 | 0.163 |
| 1087 | 21308 | 0.035 | 0.054 | 0.163 |
| 1088 | 21296 | 0.056 | 0.09 | 0.163 |
| 1089 | 21298 | 0.053 | 0.088 | 0.163 |
| 1090 | 21310 | 0.025 | 0.039 | 0.163 |
| 1091 | 21301 | 0.036 | 0.074 | 0.163 |
| 1092 | 9738 | 0.706 | 0.833 | 0.163 |
| 1093 | 20106 | 0.473 | 0.592 | 0.163 |
| 1094 | 20113 | 0.378 | 0.693 | 0.163 |
| 1095 | 9922 | 0.731 | 0.84 | 0.164 |
| 1096 | 9920 | 0.748 | 0.844 | 0.164 |
| 1097 | 9921 | 0.74 | 0.842 | 0.164 |
| 1098 | 20114 | 0.32 | 0.652 | 0.166 |
| 1099 | 9933 | 0.45 | 0.599 | 0.166 |
| 1100 | 9935 | 0.438 | 0.599 | 0.166 |
| 1101 | 9936 | 0.419 | 0.593 | 0.166 |
| 1102 | 9932 | 0.459 | 0.601 | 0.166 |
| 1103 | 9934 | 0.442 | 0.597 | 0.166 |
| 1104 | 9937 | 0.403 | 0.605 | 0.166 |
| 1105 | 9954 | 0.202 | 0.448 | 0.167 |
| 1106 | 9957 | 0.238 | 0.45 | 0.167 |
| 1107 | 9961 | 0.171 | 0.229 | 0.167 |
| 1108 | 9960 | 0.162 | 0.401 | 0.167 |
| 1109 | 9956 | 0.155 | 0.3 | 0.167 |
| 1110 | 9951 | 0.289 | 0.512 | 0.167 |
| 1111 | 9950 | 0.197 | 0.359 | 0.167 |
| 1112 | 9955 | 0.231 | 0.298 | 0.167 |
| 1113 | 4566 | 0.734 | 0.826 | 0.168 |
| 1114 | 4567 | 0.714 | 0.819 | 0.168 |
| 1115 | 4599 | 0.741 | 0.829 | 0.168 |
| 1116 | 4138 | 0.736 | 0.837 | 0.168 |
| 1117 | 9759 | 0.239 | 0.304 | 0.168 |
| 1118 | 9927 | 0.563 | 0.791 | 0.169 |
| 1119 | 9975 | 0.23 | 0.301 | 0.17 |
| 1120 | 9968 | 0.277 | 0.354 | 0.17 |
| 1121 | 9974 | 0.155 | 0.194 | 0.17 |
| 1122 | 9966 | 0.172 | 0.378 | 0.17 |
| 1123 | 9962 | 0.17 | 0.305 | 0.17 |
| 1124 | 9963 | 0.242 | 0.414 | 0.17 |
| 1125 | 9967 | 0.195 | 0.24 | 0.17 |
| 1126 | 9969 | 0.144 | 0.275 | 0.17 |
| 1127 | 20105 | 0.619 | 0.787 | 0.17 |
| 1128 | 9756 | 0.43 | 0.617 | 0.171 |
| 1129 | 9775 | 0.451 | 0.571 | 0.172 |
| 1130 | 1249 | 0.361 | 0.39 | 0.172 |
| 1131 | 1248 | 0.414 | 0.448 | 0.172 |
| 1132 | 9742 | 0.657 | 0.81 | 0.173 |
| 1133 | 9965 | 0.371 | 0.491 | 0.173 |
| 1134 | 4143 | 0.633 | 0.801 | 0.173 |
| 1135 | 4142 | 0.667 | 0.813 | 0.173 |
| 1136 | 4141 | 0.686 | 0.819 | 0.173 |
| 1137 | 4140 | 0.704 | 0.825 | 0.173 |
| 1138 | 4139 | 0.724 | 0.831 | 0.173 |
| 1139 | 20109 | 0.455 | 0.609 | 0.173 |
| 1140 | 20108 | 0.462 | 0.595 | 0.174 |
| 1141 | 20104 | 0.655 | 0.798 | 0.174 |
| 1142 | 20110 | 0.416 | 0.585 | 0.174 |
| 1143 | 4569 | 0.714 | 0.821 | 0.175 |
| 1144 | 4568 | 0.734 | 0.828 | 0.175 |
| 1145 | 1254 | 0.652 | 0.809 | 0.176 |
| 1146 | 1247 | 0.467 | 0.509 | 0.177 |
| 1147 | 20107 | 0.458 | 0.583 | 0.181 |
| 1148 | 1256 | 0.606 | 0.797 | 0.184 |
| 1149 | 1251 | 0.698 | 0.822 | 0.184 |
| 1150 | 1253 | 0.67 | 0.816 | 0.185 |
| 1151 | 1255 | 0.636 | 0.808 | 0.185 |
| 1152 | 2006 | 0.76 | 0.888 | 0.192 |
| 1153 | 2008 | 0.727 | 0.879 | 0.192 |
| 1154 | 2005 | 0.774 | 0.893 | 0.192 |
| 1155 | 2009 | 0.705 | 0.874 | 0.192 |
| 1156 | 2007 | 0.738 | 0.884 | 0.192 |
| 1157 | 1252 | 0.692 | 0.824 | 0.194 |
| 1158 | 1326 | 0.128 | 0.286 | 0.2 |
| 1159 | 60001 | 0.775 | 0.881 | 0.2 |
| 1160 | 11537 | 0.779 | 0.876 | 0.2 |
| 1161 | 11536 | 0.791 | 0.879 | 0.2 |
| 1162 | 11539 | 0.747 | 0.868 | 0.2 |
| 1163 | 11540 | 0.726 | 0.863 | 0.2 |
| 1164 | 11541 | 0.706 | 0.857 | 0.2 |
| 1165 | 11538 | 0.768 | 0.873 | 0.2 |

**Table S5. Optical properties of window glass used in EnergyPlus simulation for high-latitude regions.**

| Optical properties | Normal glass  (CLEAR 6MM) | Commercial  low-e glass^[9]^ | Our radiative warming glass |
| --- | --- | --- | --- |
| *T*_sol_ | 0.775 | 0.63 | 0.836 |
| *R*_sol-front_ | 0.071 | 0.22 | 0.054 |
| *R*_sol-back_ | 0.071 | 0.19 | 0.057 |
| *T*_vis_ | 0.881 | 0.85 | 0.877 |
| *R*_vis-front_ | 0.08 | 0.079 | 0.053 |
| *R*_vis-back_ | 0.08 | 0.056 | 0.054 |
| *ε*_front_ | 0.84 | 0.1 | 0.117 |
| *ε*_back_ | 0.84 | 0.84 | 0.84 |

**Table S6. The cost of some commercial low-e glass** **compared to this work.**

| Manufacturer | Cost  /Square meter | Model NO. |
| --- | --- | --- |
| Shandong Jinjing Science & Technology Stock Co., Ltd. | US$50.00 | 3-60mm |
| Luoyang LandGlass Technology Co., Ltd. | US$250.00-320.00 | LD-Vacuum Glass |
| Quyang Blue Ville Landscaping Sculpture Co., Ltd. | US$50.00-230.00 | GD-80 |
| Beijing Bright View Windows and Glass Co., Ltd. | US$150.00 | G55 |
| JOSHUA STARTEK LTD. | US$80.00-105.00 | PUV1 |
| Guangzhou Morrison Building Materials LLC | US$75.00 | 120 |
| Doorwin Windows Inc | US$138.00-188.00 | TN1 |
| VIET PHAP ALUMINUM FACTORY - VIET PHAP ALUMINUM JOINT STOCK COMPANY | US$90.00-100.00 | 6631 |
| This work | US4.79 |  |

**Reference**

[1] Z. Zhou, Y. Fang, X. Wang, E. Yang, R. Liu, X. Zhou, Z. Huang, H. Yin, J. Zhou, B. Hu, *Nano Energy* **2022**, *93*, 106865.

[2] Y. Chen, J. Mandal, W. Li, A. Smith-Washington, C. C. Tsai, W. Huang, S. Shrestha, N. Yu, R. P. S. Han, A. Cao, Y. Yang, *Sci. Adv.* **2020**, *6*.

[3] J. Mandal, M. Jia, A. Overvig, Y. Fu, E. Che, N. Yu, Y. Yang, *Joule* **2019**, *3*, 3088.

[4] S. K. Saju, A. B. Puthirath, S. Wang, T. Tsafack, L. K. Beagle, A. Baydin, N. Chakingal, N. Komatsu, F. Tay, A. Sharma, R. Sreenivasan, J. Kono, R. Vajtai, N. R. Glavin, Y. Long, P. M. Ajayan, *Joule* **2024**, *8*, 2696.

[5] Y. Zhu, H. Luo, C. Yang, B. Qin, P. Ghosh, S. Kaur, W. Shen, M. Qiu, P. Belov, Q. Li, *Light: Sci. Appl.* **2022**, *11*, 122.

[6] Y. Jin, Y. Jeong, K. Yu, *Adv. Funct. Mater.* **2023**, *33*, 2207940.

[7] K. Lin, S. Chen, Y. Zeng, T. C. Ho, Y. Zhu, X. Wang, F. Liu, B. Huang, C. Y. Chao, Z. Wang, C. Y. Tso, *Science* **2023**, *382*.

[8] X. Zhao, T. Li, H. Xie, H. Liu, L. Wang, Y. Qu, S. C. Li, S. Liu, A. H. Brozena, Z. Yu, J. Srebric, L. Hu, *Science* **2023**, *382*.

[9] S. Wang, T. Jiang, Y. Meng, R. Yang, G. Tan, Y. Long, *Science* **2021**, *374*, 1501.

[10] C. Lin, J. Hur, C. Y. H. Chao, G. Liu, S. Yao, W. Li, B. Huang, *Sci. Adv.* **2022**, *8*, eabn7359.

[11] C. Sui, J. Pu, T.-H. Chen, J. Liang, Y.-T. Lai, Y. Rao, R. Wu, Y. Han, K. Wang, X. Li, V. Viswanathan, P.-C. Hsu, *Nat. Sustain.* **2023**, *6*, 428.

[12] Y. Jia, D. Liu, D. Chen, Y. Jin, C. Chen, J. Tao, H. Cheng, S. Zhou, B. Cheng, X. Wang, Z. Meng, T. Liu, *Nat. Commun.* **2023**, *14*.

[13] Y. Rao, J. Dai, C. Sui, Y.-T. Lai, Z. Li, H. Fang, X. Li, W. Li, P.-C. Hsu, *ACS Energy Lett.* **2021**, *6*, 3906.

[14] Y. Ke, Y. Li, L. Wu, S. Wang, R. Yang, J. Yin, G. Tan, Y. Long, *ACS Energy Lett.* **2022**, *7*, 1758.

[15] S. Lin, H. Wang, X. Zhang, D. Wang, D. Zu, J. Song, Z. Liu, Y. Huang, K. Huang, N. Tao, Z. Li, X. Bai, B. Li, M. Lei, Z. Yu, H. Wu, *Nano Energy* **2019**, *62*, 111.

[16] K. Xu, M. Du, L. Hao, J. Mi, Y. Lin, S. Li, J. Wang, X. Deng, *Infrared Phys. Technol.* **2022**, *122*, 104089.

[17] M. L. Addonizio, M. Ferrara, A. Castaldo, A. Antonaia, *Energy Build.* **2021**, *250*, 111259.

[18] H. Hu, S. Wang, Y. Meng, G. Liu, M. Li, T. D. Vu, Y. Long, *Adv. Mater. Technologies* **2021**, *7*, 2100824.

[19] K. Sun, X. Tang, C. Yang, D. Jin, *Ceram. Int.* **2018**, *44*, 19597.
